# Characterization of CD4^+^ and CD8^+^ T cells responses in the mixed lymphocyte reaction by flow cytometry and single cell RNA sequencing

**DOI:** 10.3389/fimmu.2023.1320481

**Published:** 2024-01-12

**Authors:** Adèle Mangelinck, Agathe Dubuisson, Etienne Becht, Sandra Dromaint-Catesson, Manon Fasquel, Nicolas Provost, Dawid Walas, Hélène Darville, Jean-Pierre Galizzi, Céline Lefebvre, Véronique Blanc, Vincent Lombardi

**Affiliations:** Servier, Research and Development, Gif-sur-Yvette, France

**Keywords:** mixed lymphocyte reaction, T cells, immuno-oncology, immune checkpoint, single cell RNA sequencing, cell-cell communication

## Abstract

**Background:**

The Mixed Lymphocyte Reaction (MLR) consists in the allogeneic co-culture of monocytes derived dendritic cells (MoDCs) with T cells from another donor. This *in vitro* assay is largely used for the assessment of immunotherapy compounds. Nevertheless, the phenotypic changes associated with lymphocyte responsiveness under MLR have never been thoroughly evaluated.

**Methods:**

Here, we used multiplex cytokine and chemokine assays, multiparametric flow cytometry and single cell RNA sequencing to deeply characterize T cells activation and function in the context of CD4^+^- and CD8^+^-specific MLR kinetics.

**Results:**

We showed that CD4^+^ and CD8^+^ T cells in MLR share common classical markers of response such as polyfunctionality, increased proliferation and CD25 expression but differ in their kinetics and amplitude of activation as well as their patterns of cytokines secretion and immune checkpoints expression. The analysis of immunoreactive Ki-67^+^CD25^+^ T cells identified PBK, LRR1 and MYO1G as new potential markers of MLR response. Using cell-cell communication network inference and pathway analysis on single cell RNA sequencing data, we also highlighted key components of the immunological synapse occurring between T cells and the stimulatory MoDCs together with downstream signaling pathways involved in CD4^+^ and CD8^+^ T cells activation.

**Conclusion:**

These results provide a deep understanding of the kinetics of the MLR assay for CD4^+^ or CD8^+^ T cells and may allow to better characterize compounds impacting MLR and eventually identify new strategies for immunotherapy in cancer.

## 1 Introduction

Cancer results from abnormal proliferation of cells evading immunosurveillance. This phenomenon relies on complex suppressive mechanisms leading to the inhibition of immune cells at the tumor site (1–3). Restoring the capacity of immune effector cells, especially T cells, to recognize and eliminate cancer cells is the goal of immunotherapy.

Over the past decade, immunotherapy has been a real breakthrough in cancer treatment. In particular, antibodies targeting inhibitory immune checkpoints (ICPs), such as anti-programmed death-1 (PD-1) and anti-cytotoxic T-lymphocyte-associated protein 4 (CTLA-4), have drastically transformed the therapeutic strategies for a wide range of tumors (4). However, the underlying mechanisms of action of ICPs have not been fully deciphered yet (5). Consequently, there is a need to deeply characterize the pre-clinical immune assays that allowed to evaluate them in order to better understand the functions of these ICPs (6).

The Mixed Lymphocyte Reaction (MLR) is an *in vitro* cellular immune assay that was first described by Bain et al. in 1963 (7). It is performed to evaluate T cell responsiveness to “non-self” antigen presenting cells. As such, MLR represents a powerful tool for the assessment of new immunotherapies. Notably, this assay has been used for the *in vitro* evaluation of Nivolumab activity (8). Nivolumab is an anti-PD-1 monoclonal antibody (mAb) that is indicated for the treatment of a large panel of solid tumors as well as hematological malignancies (9). PD-1, along with other ICPs, has been shown to be stably overexpressed upon chronic T cell activation and is in part responsible for the acquisition of an altered phenotype called exhaustion (10–13). In the context of allogeneic transplantation, overexpression and agonism of inhibitory ICPs signaling pathways led to allograft tolerance, whereas absence or blockade led to accelerated rejection and lethality in mice (14–16). A large number of pre-clinical studies have shown that targeting inhibitory ICPs can restore and/or enhance T cell effector functions (17–19). However, inhibitory ICPs expression has also been reported to be induced upon allogeneic stimulation as early as the acute phase of T cell activation and are thus not specific to exhaustion state or phenotype (20–24).

A previous study evaluated ICPs expression and cytokine production at median and late phases of a MLR assay (25). They focused on PD-1, LAG-3 and TIM-3 inhibitory ICPs and showed that their expression patterns differed from CD4^+^ to CD8^+^ T cells upon allogeneic stimulation by monocyte-derived dendritic cells (MoDCs). Nonetheless, phenotypic changes associated with lymphocyte responsiveness under MLR have never been completely characterized. Here, we describe CD4^+^- and CD8^+^-specific MLR assays with a deep characterization based on large panels of cytokines, assessment of inhibitory and activatory ICPs by flow cytometry and single cell RNA sequencing (scRNA-seq) at early and late timepoints. We confirmed that T cell activation and function in the MLR concur with different patterns of cytokines production in CD4^+^ and CD8^+^ T cells. We also confirmed that the frequency of proliferating and activated Ki-67^+^CD25^+^ T cells increases in MLR. Thanks to single cell RNA-seq data, we identified this Ki-67^+^CD25^+^ population as T helper cells (Thelper) and regulatory T cells (Treg) for CD4^+^ T cells and cytotoxic T cells (Tcyt) for CD8^+^ T cells and found new potential markers of MLR-reactivity. We then demonstrated that these immunoreactive T cells co-expressed both inhibitory and activatory ICPs and that this co-expression correlated with cytokine secretion. Further cell-cell communication network inference and pathway analysis based on single cell RNA-seq data highlighted key components of the immunological synapse that occurs between MoDCs and T cells, and downstream signaling pathways involved in early and late phases of CD4^+^ and/or CD8^+^ T cell activation.

## 2 Materials and methods

### 2.1 Isolation of primary cells (PBMC, CD14^+^ monocytes, CD4^+^ and CD8^+^ T cells)

Blood samples from 4 healthy donors were purchased from Etablissement Français du Sang (EFS, Pontoise, France). Peripheral blood mononuclear cells (PBMCs) were isolated from buffy coat by density gradient centrifugation using Ficoll : Lymphoprep (cat no. 07801, StemCell). CD14^+^, CD4^+^ and CD8^+^ populations were purified from PBMCs using magnetic CD14 isolation beads by positive selection (CD14 Microbeads human, cat no. 130-050-201, Miltenyi) and CD4 (CD4 T Cell Isolation kit, cat no. 130-096-533, Miltenyi) or CD8 (CD8 T Cell Isolation kit, cat no. 130-096-495, Miltenyi) isolation beads by negative selection, respectively, following the manufacturer’s instructions. Monocytes were isolated from 2 healthy donors (A1 and A2) and CD4^+^ as well as CD8^+^ T cells from 2 other distinct donors (B1 and B2).

### 2.2 MoDC generation from CD14^+^ cells

Monocyte-derived Dendritic cells (MoDCs) were generated by culturing CD14^+^ monocytes (10^6^/mL) *in-vitro* for 7 days in complete medium: RPMI 1640 GlutaMAX (cat no. 61870, Gibco) supplemented with 10% heat inactivated serum (cat no. CVSVF00-01, Eurobio), 1% PS (cat no. 15140, Gibco), HEPES 10 mM (cat no. 15630, Gibco), in the presence of 50 ng/mL interleukin-4 (IL-4, cat no. 130-093-922, Miltenyi) and 100 ng/mL GM-CSF (cat no. 130-093-866, Miltenyi) (26).

After 7 days, and prior to co-culture, MoDCs were tested for differentiation and maturation status by measuring CD1a, CD83, CD86, Tim-3, PD-L1 and HLA-DR expression by flow cytometry.

### 2.3 One-way mixed lymphocyte reaction kinetic

Freshly isolated CD4^+^ or CD8^+^ T cells (10^5^ cells) and allogeneic MoDCs (10^4^ cells) were co-cultured in triplicate in culture media consisting of RPMI 1640 no Glucose (cat no. 11879, Gibco) supplemented with 11mM D-Glucose (cat no. G8769, Sigma), 10% heat inactivated serum (cat no. CVSVF00-01, Eurobio) and 1% P/S (cat no. 15140, Gibco), using an ultra-low attachment 96-well microplate (Corning, cat no. 7007). As controls, CD4^+^ or CD8^+^ T cells were cultured alone or in combination with anti-CD3/anti-CD28 antibodies (ImmunoCult™ Human CD3/CD28 T Cell Activator, cat no. 10971, StemCell) in triplicate in culture media.

**Figure 1:**
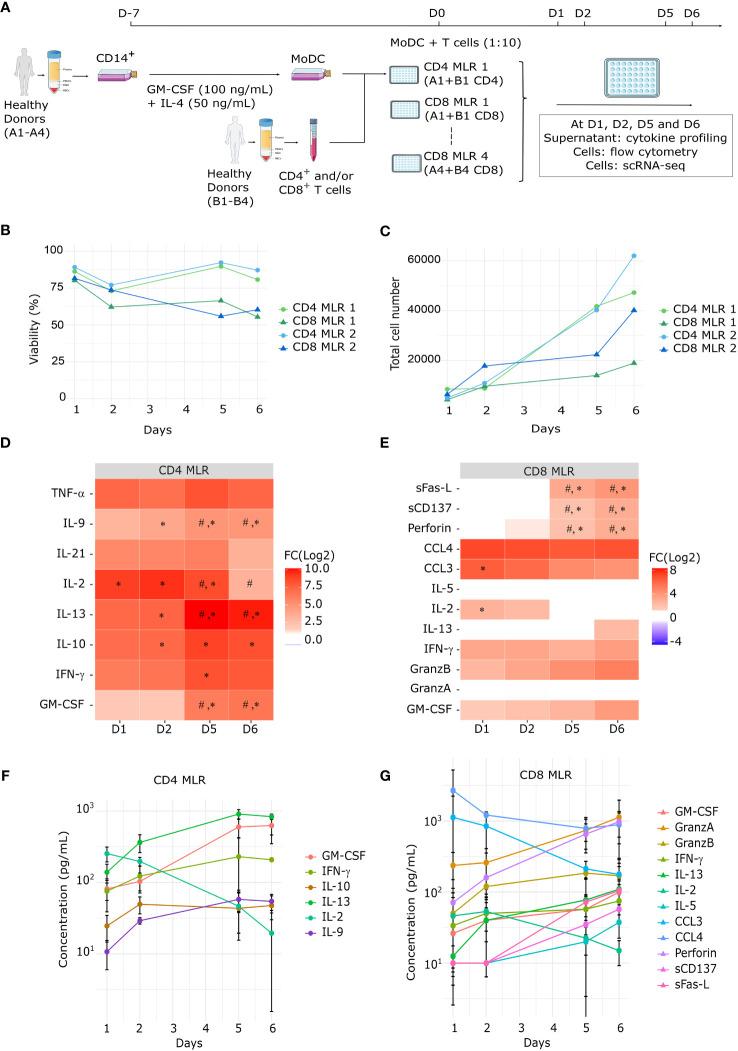
Kinetic of polyfunctional cytokine release upon MLR stimulation. **(A)** Schematic representation of the MLR protocol **(B, C)** Time-course evaluation by flow cytometry of MLR cell viability **(B)** and total cell number **(C)** of four MLRs (1 to 4, n=4). **(D, E)** Heatmap of the fold change for each soluble factor between MLR stimulation vs T cells alone at each timepoint for **(D)** all CD4 (n=4) or **(E)** all CD8 (n=4) MLRs (1 to 8). Statistical analyses: ANOVA with Tukey’s multiple comparisons tests, #: p-value < 0.05 for the corresponding soluble factor between MLR-stimulated cells at D1 and MLR-stimulated cells at the indicated day; *: p-value < 0.05 for the corresponding soluble factor between non-stimulated cells and MLR-stimulated cells on the same day. **(F, G)** Time-course concentrations of secreted cytokines in CD4 **(F)** or CD8 **(G)** MLRs. Each dot and bars represent the mean and standard deviation (SD) in MLR-stimulated T cells (n=4) for each soluble factor at the corresponding time.

Eight different MLRs were performed using 4 different donors (CD4 MLR: n=4; CD8 MLR: n=4): (1) MoDCs from A1 co-cultured with CD4^+^ T cells from B1, (2) MoDCs from A1 co-cultured with CD8^+^ T cells from B1, (3) MoDCs from A2 co-cultured with CD4^+^ T cells from B2, (4) MoDCs from A2 co-cultured with CD8^+^ T cells from B2, (5) MoDCs from A3 co-cultured with CD4^+^ T cells from B3, (6) MoDCs from A3 co-cultured with CD8^+^ T cells from B3, (7) MoDCs from A4 co-cultured with CD4^+^ T cells from B4 and (8) MoDCs from A4 co-cultured with CD8^+^ T cells from B4 (see **Figure 1A**).

On day 1, 2, 5 and 6, culture supernatants from all MLRs (1 to 8) and cells from four MLRs (1 to 4) were collected. Culture supernatants were immediately frozen at -80°C for at least 24h, until further analyses. Cells were resuspended and collected for either flow cytometry (3x10^5^ cells) or scRNA-seq (9x10^5^ cells) analyses. For scRNA-seq, cells were collected at day 1, 2, 5 and 6, and CD4^+^ or CD8^+^ T cells alone were collected at day 1 and day 5.

### 2.4 Flow cytometry

MoDCs were tested for maturation status using anti-CD14-AF488 (clone: M5E2, Biolegend), anti-CD3-PercP (clone: UCHT1, Biolegend), anti-CD1a-APC (clone: HI149, Miltenyi), anti-CD83-BV605 (clone: HB15, Biolegend), anti-CD86-PE/Vio770 (clone: FM95, Miltenyi), anti-HLA-DR-BV610 (clone: L243, Biolegend), anti-Tim-3-BV711 (clone: F38-E2E, Biolegend), anti-OX40-L-PE (clone: ANC10G1, Ancell), anti-PD-1-BV421 (clone: EH12.2H7, Biolegend), anti-PD-L1-PECF594 (clone: 2A3, Biolegend) antibodies. Viability was assessed using Zombie NIR (cat no. 423106, Biolegend). Acquisition and analyses were performed on CytoFLEX S (Beckman Coulter).

At day 1, 2, 5 and 6 after co-culture, cells from four MLRs (1 to 4) were labeled to measure T cell proliferation and activation status. Fc receptor blocking was done on the total cell suspension using human FcR Blocking reagent (cat no. 130-059-901, Miltenyi). Cell viability was determined using Maleimide (cat no. 1408, AAT Bioquest). For cell surface phenotyping, anti-CD3-APC-Cy7, anti-CD4-BUV496, anti-CD8-PerCP, anti-CCR7-BV510, anti-CD45RA-BV805, anti-CD25-FITC, anti-CTLA-4-PECF594, anti-PD-1-BUV737, anti-Tim-3-BV785, anti-LAG-3-BV421, anti-ICOS-AF700, anti-TIGIT-BUV595, anti-NKG2A-PC7 were used. For intracellular phenotyping: cells were fixed and permeabilized using human FoxP3/Transcription Factor Staining Buffer Set (cat no. 00-5523-00, Thermo Fisher Scientific) according to the manufacturer’s direction and stained with anti-FoxP3-PE (clone: PCH101, eBioscience), anti-TOX-APC (clone: TXRX10, eBioscience) and Ki67-BV711 (cat no. 350516, BioLegend). Cells were acquired on CytoFLEX LX (Beckman Coulter). Data visualization and analysis were performed using Kaluza Analysis software v2.1 (Beckman Coulter), Cytobank software (Beckman Coulter) and R v3.6.1.

### 2.5 Multiplex cytokine and chemokine assays

Cytokine and chemokine concentrations in culture supernatants from all MLRs (1 to 8) were evaluated in triplicate using Milliplex MAP Human TH17 kit (Merck, cat no. HCYTOMAG-60K) for CD4^+^ T cells and Milliplex MAP Human CD8 kit (Merck, cat no. HC8MAG-15K-13) for CD8^+^ T cells.

Acquisitions and analyses were performed on a Bio-Plex 200 system (Bio-Rad) and a Bio-Plex Manager 6.1 Software (Bio-Rad), respectively. Data visualization was performed in R v3.6.1 using dplyr (v2.3.2), tidyr (v1.3.0), ggpubr (v0.4.0), or Hmisc (v4.4.1) packages. Fold change (FC) represents the ratio between the mean of each cytokine and chemokine concentration in MLR-stimulated (medium) condition over non-stimulated cells. All calculations were performed using R v3.6.1.

### 2.6 Single cell RNA sequencing

At each timepoint (D1, D2, D5 and D6), 9x10^5^ live cells were recovered from four MLRs (1 to 4). After being washed once with 0.04% BSA in 1X PBS and centrifuged at 300g for 5 minutes, cells were processed through 10x Cell Multiplexing Oligo Labeling protocol (10x Genomics, USA) according to the manufacturer’s instructions. Pooled cell suspensions (~1,500 cells/µl) were prepared with equal number of cells per sample, one for MLR samples and one for T cells samples at each time point. Due to lower cell viability, CD8 samples from donor 2 MLR were processed separately without multiplexing labeling to prepare ~1,000 cells/µl cell suspensions. Respectively, 23,100-8,250 cells were used for the 10x Chromium Single-Cell 3’ v3.1 protocol with/without Feature Barcode (10x Genomics, USA), according to the manufacturer’s instructions. Libraries were sequenced on a NovaSeq 6000 sequencer (Illumina, USA).

### 2.7 Single cell RNA sequencing data analysis

Cell Ranger (v6.0.1, 10x Genomics Inc) was applied for demultiplexing, reads mapping against the GRCh38 human reference genome, and UMI counting. Then, the Seurat package (v4.2.1) was used to generate Seurat objects. Only genes detected in at least 3 cells were kept. Cells with fewer than 200 genes detected or >15% mitochondrial UMI counts were filtered out. Samples were merged in a CD4 and a CD8 objects according to their T cell type then count data normalization and scaling was performed using Seurat with default parameters. Genes were ranked descendingly by residual variance estimated from the “vst” method implemented in the *FindVariableFeatures* function from Seurat. Excluding immunoglobulin, ribosome-protein-coding, and T cell receptor (TCR) genes (gene symbol with string pattern “^IGK|^IGH|^IGL|^IGJ|^IGS|^IGD|IGFN1”, “^RP([0-9]+-|[LS])”, and “^TRA|^TRB|^TRG” respectively), the top 2000 genes were identified as highly variable genes and used for Principal Component Analysis (PCA). Harmony (27) (v0.1.1) was applied for batch effect correction then Uniform Manifold Approximation and Projection (UMAP) and clustering using the Louvain algorithm were performed on the harmony reduction. Non-T cell or -MoDC clusters were removed for further analysis.

Additional R packages used alongside the scRNA-seq analysis are tidyverse (v1.3.2), ggpubr (v0.4.0), gridExtra (v2.3), cowplot (v1.1.1), xlsx (v0.6.5), ComplexHeatmap (28) (v2.12.1), circlize (v0.4.15), viridis (v0.6.2) and lemon (v0.4.6).

### 2.8 Differential expression and pathway analysis

For the transcriptomic characterization of the MKI67^+^IL2RA^+^ population, the *FindAllMarkers* function from Seurat was used with the batch effect variable specified in the latent.vars, *MAST* method and default thresholds. We further filtered out genes for which the adjusted p-value was superior or equal to 0.05.

For the study of signaling pathways involved in early and late phases of CD4^+^ and/or CD8^+^ T cell activation, scRNA-seq count matrices were aggregated to the sample level on T cells subsets using the *AggregateExpression* function from Seurat. Then, DESeq2 (29) (v1.38.3) was run with default parameters using the paired T cells control at day 1 as a reference to identify differentially expressed genes. Resulting p-values were adjusted with the alpha parameter set to 0.05 for multiple test correction and log2 fold changes were corrected by shrinkage estimation with the *lfcShrink* function and the *apeglm* method (30).

Gene Set Enrichment Analysis (GSEA) was performed using the fgsea package (31) (v1.24.0) with the annotation information of gene sets downloaded from MSigDB. The *fgsea* function was used with parameters set as follows: minSize = 15, maxSize = 500, nperm = 1000.

### 2.9 Cell-cell communication analysis

Cell-cell communication network inference was performed using the CellChat package (32) (v1.5.0) with default parameters and the human CellChatDB. One cellchat object was created for each time point and condition to perform individual analysis before merging in one object for CD4^+^ T cells and one for CD8^+^ T cells for comparative analysis.

### 2.10 Statistical analysis

For Multiplex Cytokine and Chemokine Assays, one-way analysis of variance (ANOVA) with Tukey’s multiple comparisons tests were performed per T cell type (CD4 MLR: n=4; CD8 MLR: n=4) using the multcomp package (v1.4-13) for multiple group comparisons. #: p-value < 0.05 for the corresponding soluble factor between MLR-stimulated cells at D1 and MLR-stimulated cells at the indicated day; *: p-value < 0.05 for the corresponding soluble factor between non-stimulated cells and MLR-stimulated cells on the same indicated day.

## 3 Results

### 3.1 CD4^+^ and CD8^+^ T cells undergo polyfunctional activation and differential proliferation in MLR

To decipher the kinetics of CD4^+^ and CD8^+^ T cell responses in allogeneic one-way MLR, we conducted four sets of separated CD4 and CD8 MLRs. For each biological replicate, CD4^+^ T cells and CD8^+^ T cells from the same donor were cultured separately with MoDCs from another donor (**Figure 1A**). Cells and supernatants from these 8 MLRs were harvested after 1, 2, 5 or 6 days. At each timepoint, cell viability and numbers were measured by flow cytometry for four MLRs (1 to 4) (**Figures 1B, C**). In both CD4 and CD8 MLRs, whereas viability remained broadly stable over time, total cell number showed an expansion between day 2 and day 6. Notably, T cell viability and expansion were substantially lower in CD8 MLRs compared to CD4 MLRs, irrespective of the donors.

Cytokine production was evaluated by a multiplex cytokine assay on day 1, 2, 5 and 6 in all MLRs (1 to 8). Most of soluble immune factors comprised in the panels showed increased concentrations in MLR compared to control non-stimulated T cells (**Figures 1D, E**). Most cytokines were secreted from day 1 and accumulated over time: IFN-γ, GM-CSF, IL-9, IL-10, IL-13 for CD4 MLRs, and GzmB, GM-CSF, Perforin for CD8 MLRs (**Figures 1F, G**; **Supplementary Figures S1A**, **S2A**). Notably, sCD137 and sFasL secretions were only detected after day 2 in CD8 MLRs. As expected, the diversity of secreted cytokines indicates that MLR induces polyfunctional responses not limited to Th1 (IFN-γ) or Tc1 (GzmB) cytokines (25, 33–35). Interestingly, the levels of IL-2 in the supernatants decreased over time in both CD4 and CD8 MLRs. IL-2 being a T cell growth factor (36–39), its decrease observed during MLR is therefore probably due to its consumption by T cells. Moreover, even though cell expansion was higher in CD4 MLRs compared to CD8 MLRs, cytokines common to the CD4 and CD8 panels appeared to show the same range of increase. Hence, CD4^+^ and CD8^+^ T cells in MLR are capable of mounting polyfunctional responses but CD4^+^ T cells expand more efficiently.

### 3.2 CD8^+^ T cells activation is delayed in MLR compared to CD4^+^ T cells

**Figure 2:**
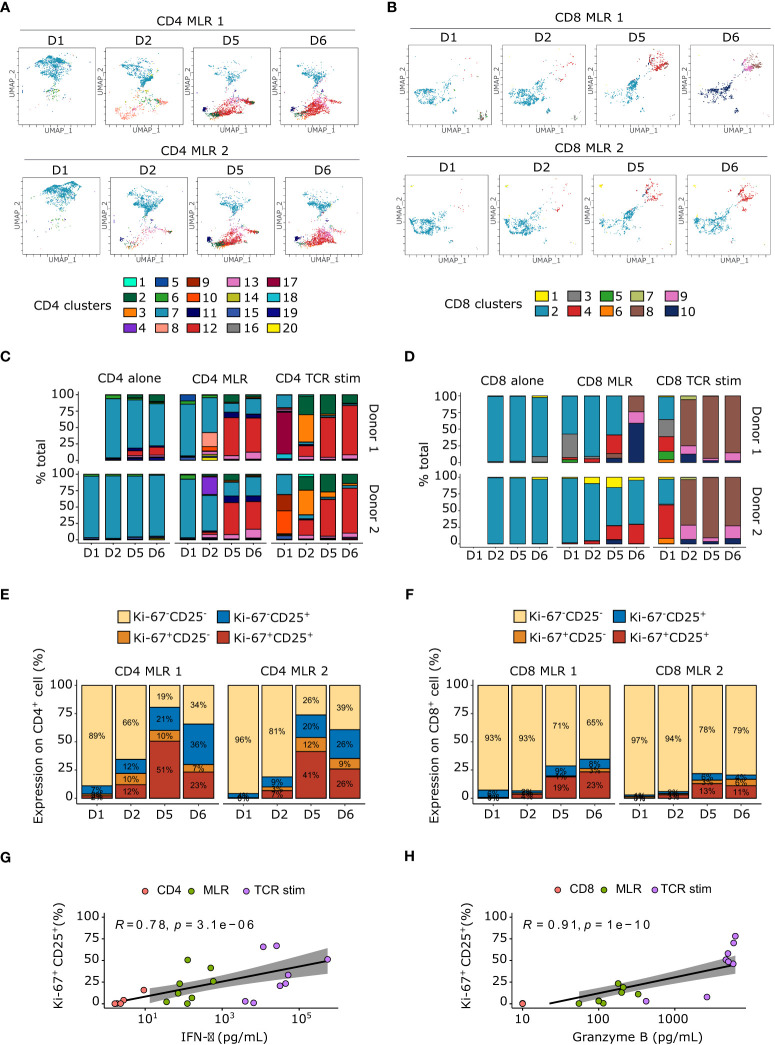
Immunoreactive Ki-67^+^CD25^+^ cell populations increase over time upon MLR stimulation. **(A, B)** Flow cytometry UMAP dimensionality reduction representation of CD4^+^ **(A)** and CD8^+^ **(B)** T cells upon MLR stimulation colored by non-supervised clustering. **(C, D)** Barplot of cell proportions colored by non-supervised clustering for CD4^+^ **(C)** or CD8^+^ T cells. **(E, F)** Barplot of Ki-67 and CD25 expression upon CD4 **(E)** or CD8 **(F)** MLRs. **(G)** Spearman correlation between IFN-γ secretion and percentage of Ki-67^+^CD25^+^ in CD4^+^ T cells. **(H)** Spearman correlation between granzyme B secretion and percentage of Ki-67^+^CD25^+^ in CD8^+^ T cells. TCR stim: T cells stimulated with anti-CD3 and anti-CD28 antibodies.

To investigate the phenotypic specificities of CD4 and CD8 MLRs over time, we conducted UMAP dimensionality reduction and unsupervised clustering of a multiparametric flow cytometry panel (18-color). Cells separated into 20 clusters for CD4 MLRs (**Figure 2A**; **Supplementary Figure S1B**) and 10 clusters for CD8 MLRs (**Figure 2B**; **Supplementary Figure S2B**), based on expression levels of the studied protein markers. We observed that non-stimulated T cells clustered all together in cluster 7 for the CD4^+^ T cells and cluster 2 for the CD8^+^ T cells. More generally, cells in cluster 7 for the CD4^+^ T cells and cluster 2 for the CD8^+^ T cells expressed only memory or naïve markers, *i.e.* CCR5 and/or CD45RA (**Figures 2C, D**). Therefore, they correspond to non-activated T cells. In contrast, T cells that received a T cell receptor (TCR)-specific stimulation by anti-CD3 and anti-CD28 antibodies mainly clustered in cluster 12 for the CD4^+^ T cells and cluster 8 for the CD8^+^ T cells at day 5 and 6. Upon MLR stimulation, cluster 12 for the CD4^+^ T cells and clusters 4 and/or 8 for the CD8^+^ T cells were also enriched. Of note, for the CD8^+^ T cells, cluster 8 represents highly activated cells (with high expression of all activated markers) whereas cluster 4 appears for medium activation state. As these clusters expressed medium to high levels of CD25, Ki-67, PD-1, ICOS, Tim-3, we called them “immunoreactive clusters”. These immunoreactive clusters increased over time and became predominant at day 5 and 6 (from 2.07 +/- 1.48% at day 2 to 53.2 +/- 4.6% at day 5 in CD4 MLRs) whereas the non-activated T cells clusters decreased over the course of stimulation (from 53.6 +/- 0.5% at day 2 to 17.3 +/- 3.7% at day 5 in CD4 MLRs). We observed differences in responsiveness between CD4^+^ and CD8^+^ T cells in MLR. In CD8 MLRs, the immunoreactive cluster only appeared from day 5 and represented 24.1 +/- 3.6% while the non-activated population still constituted 57.7 +/- 0.6% of total cells at this late timepoint. Hence, the CD8^+^ T cells response appears to be delayed in the MLR assay compared to CD4^+^ T cells.

These immunoreactive clusters express the classical activation and proliferation markers, CD25 and Ki-67 respectively (40, 41), whereas the non-activated clusters did not (**Supplementary Figures S1B**, **S2B**). Notably, while nearly all T cells in immunoreactive clusters expressed high levels of both CD25 and Ki-67 in CD8 MLRs, some cells in CD4 MLRs displayed a Ki-67^-^CD25^+^ phenotype. The kinetics of CD4^+^ and CD8^+^ T cells activation and proliferation were thus further explored with a co-expression analysis of these two markers (**Figures 2E, F**). Interestingly, we showed that the Ki-67^+^CD25^+^ population accumulated over time in both CD4^+^ T cell and CD8^+^ T cell MLRs. However, Ki-67^+^CD25^+^ cells are much more represented in CD4 MLR (46 +/- 5% and 16+/-3% at day 5 respectively in CD4 and CD8 MLRs). Interestingly, the percentage of Ki-67^+^CD25^+^ cells correlated with IFN-γ for the CD4 MLR and GzmB for the CD8 MLR (**Figures 2G, H**).

In CD4 MLRs only, we also identified a cluster 11 expressing high levels of CD25, FoxP3 and Ki-67 (**Supplementary Figure S1B**), thus phenotypically defining CD4^+^ Treg cells. Strikingly, this CD4^+^FoxP3^+^CD25^hi^ Treg cluster accumulated over time in the MLR and was nearly absent after anti-CD3/anti-CD28 stimulation only (9.16 +/- 1.32% in MLR *versus* 0.45 +/-0.04% in TCR stimulation at day 5). This highlights that in total CD4 MLR, Tregs are activated and expand.

Together, we showed that CD25 and Ki-67 co-expression is a good marker of both CD4^+^ and CD8^+^ T cells responsiveness in MLR. Interestingly, CD4^+^ T cells seemed to be more immunoreactive in this setup.

### 3.3 Ki-67^+^CD25^+^ T cells mainly correspond to CD4^+^ Treg and Thelper cells, and CD8^+^ Tcyt cells and show increased gene expression of PBK, LRR1 and MYO1G

**Figure 3:**
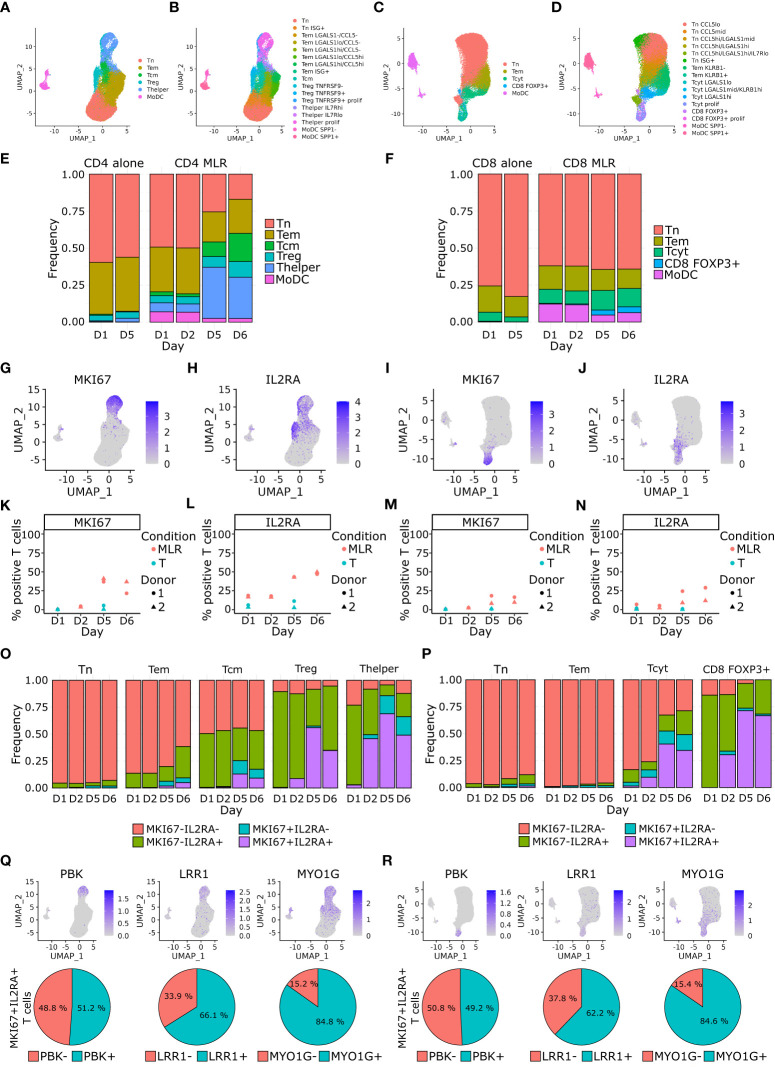
CD4^+^ Tcm, Treg and Thelper cells, and CD8^+^ Tcyt cells proportions increase over time upon MLR stimulation and acquire a proliferative phenotype. **(A–D)** Single cell RNA-seq UMAP representation of cells main **(A)** and detailed phenotypes **(B)** in the CD4 MLR, and CD8 MLR **(C, D)**. **(E, F)** Barplot of cells proportions colored by main phenotype in the CD4 MLR **(E)**, and CD8 MLR **(F)**. **(G–J)** UMAP representation of RNA expression for the indicated genes in the CD4 MLR **(G, H)**, and CD8 MLR **(I, J)**. **(K–N)** Follow up of the percentage of positive T cells for the indicated genes in the CD4 MLR **(K, L)**, and CD8 MLR **(M, N)**. **(O, P)** Barplot of MKI67-IL2RA co-expression proportions by main phenotype in the CD4 MLR **(O)**, and CD8 MLR **(P)**. **(Q, R)** UMAP representation of RNA expression (upper panel) and percentage of positive MKI67^+^IL2RA^+^ T cells (lower panel) for indicated genes in the CD4 MLR **(Q)**, and CD8 MLR **(R)**.

We further characterized immune cells in CD4 and CD8 MLRs by performing a scRNA-seq profiling at different timepoints of the MLR (namely day 1, day 2, day 5, and day 6) as well as paired T cells at early (day 1) and late (day 5) timepoints for 2 donors. After quality-control filtering, we obtained 38,624 and 37,920 cells respectively for CD4 and CD8 MLRs in total. Following integration, cells separated into 17 clusters for CD4 MLRs and 16 clusters for CD8 MLRs. These clusters could be assigned to previously described T cells and MoDCs subtypes based on both differential gene expression and interrogation of known gene markers expression (42–44) (**Figures 3A–D**; **Supplementary Figure S3**). In particular, in both CD4 and CD8 MLRs, we identified 2 subtypes of MoDCs that mainly separated on SPP1, APOE and CD68 expression. Among the CD4^+^ T cells, we identified 2 subtypes of naïve T cells (Tn) that separated on interferon-related genes expression, 6 subtypes of effector memory T cells (Tem) that separated on activation-related genes, a central memory T cells (Tcm) population, 3 subtypes of regulatory T cells (Treg) that separated on activation-related genes and 3 subtypes of T helper cells (Thelper) that separated on activation-related genes. Among the CD8^+^ T cells, we identified 6 subtypes of Tn, 2 subtypes of Tem, 4 subtypes of cytotoxic T cells (Tcyt) (all separating on activation-related genes) and a rare CD8^+^FOXP3^+^ population.

Cell composition analysis showed an increase in the Tcm, Treg and Thelper populations in CD4 MLRs and the Tcyt population in CD8 MLRs at late timepoints (**Figures 3E, F**; **Supplementary Figure S4**). To check whether the Ki-67^+^CD25^+^ T cells identified by flow cytometry could correspond to these T cells subtypes, we examined the expression of the corresponding MKI67 and IL2RA genes (**Figures 3G–J**). We confirmed that the expression of these two genes in T lymphocytes increased over time in CD4 and CD8 MLRs (**Figures 3K–N**). We then performed a detailed analysis of MKI67 and IL2RA expression by T cell subtypes. This showed that IL2RA is characteristic of Treg, Thelper and, to a lesser extent, Tcm in the CD4 MLR (more than 75% of IL2RA-expressing Tregs and Thelpers while around 50% in Tcm) without changes in the IL2RA-expressing proportions over time in all these T cell subtypes. Hence, the time-dependent increase in IL2RA expression in the CD4 MLR is a consequence of changes in T cells subtypes proportions. On the contrary, the percentage of MKI67-expressing cells progressively increased in these same CD4^+^ T cell subtypes over time (**Figure 3O**). Hence, the global increase in MKI67 expression in the CD4 MLR results from the acquisition of a proliferative phenotype by Treg, Thelper and, to a lesser extent, Tcm. In the CD8 MLR, expression of IL2RA and MKI67 increased specifically in the Tcyt and the lowly represented CD8 FOXP3+ populations (**Figure 3P**). Hence, Tcyt activation is characterized by increased expression of both IL2RA and MKI67 in this context. Single cell RNA-seq analysis therefore consistently demonstrated that immunoreactive T cells (expressing IL2RA and MKI67) found in CD4 and CD8 MLRs correspond to described subtypes of effector T cells: Treg, Thelper and Tcyt.

With the aim of identifying new potential markers of T cell responsiveness in the MLR assay, we performed an analysis of differentially expressed genes depending on MKI67 and IL2RA expression. To avoid cell population-specific genes, we ran this analysis on separated T cell subtypes. Within the Treg, Thelper and Tcyt populations, the MKI67^-^IL2RA^+^ cells showed respectively 192, 265 and 186 up-regulated genes enriched in interferon signaling pathway-related genes (**Table S1**). As for the MKI67^+^IL2RA^+^ cells, they showed respectively 1,078, 409 and 1,099 up-regulated genes (**Table S1**). In these 3 MKI67^+^IL2RA^+^ signatures, according to the adjusted p-value, MKI67 was the top differentially expressed gene and IL2RA was ranked from top 2 in the Thelper and Tcyt signatures to top 1,011 in the Treg signature. The MKI67^+^IL2RA^+^ Treg, Thelper and Tcyt signatures shared 374 genes mainly representing proliferation-, DNA replication-, mitotic spindle and microtubule organization- and mRNA processing-related genes. Among these 374 genes, we could also identify genes specifically involved in T cell activation: PBK, LRR1 and MYO1G (**Figures 3Q, R**). T cells expressing PBK, LRR1 or MYO1G represented around 50%, 65% and 85% respectively of MKI67^+^IL2RA^+^ T cells in both CD4 and CD8 MLRs. No specific activation-related genes were found to differentiate between CD4 MLR and CD8 MLR. Consequently, we showed for the first time that PBK, LRR1 and MYO1G could represent new potential markers of both CD4^+^ and CD8^+^ T cells activation in MLR besides classical proliferation markers.

### 3.4 ICPs expression increases over time in MLR and their co-expression correlates with cytokine production

**Figure 4:**
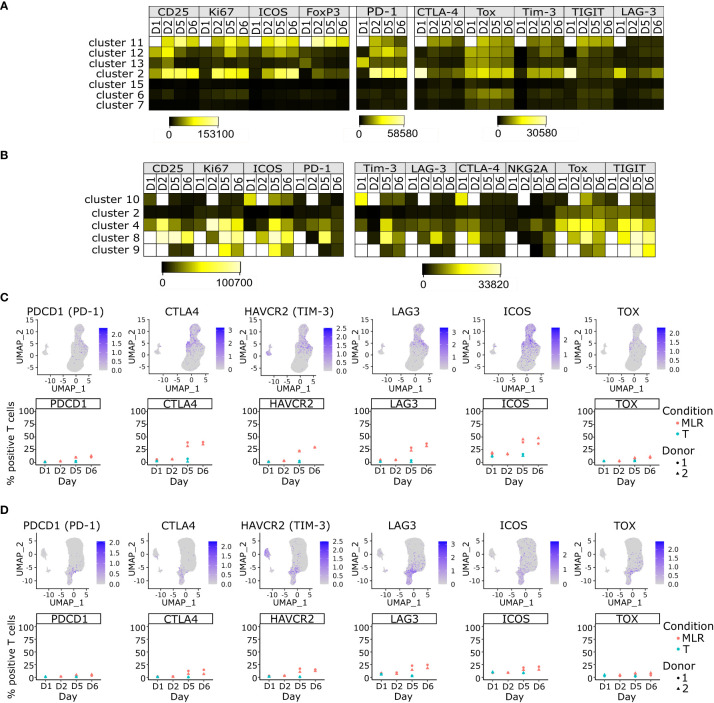
ICPs are mainly expressed by immunoreactive T cells and their expressions increase over time upon MLR stimulation. **(A, B)** Heatmap of the mean fluorescent intensity of each indicated marker per non-supervised clustering over time upon MLR-stimulation in the CD4 MLR **(A)**, or CD8 MLR **(B)** **(C, D)** UMAP representation of RNA expression and follow up of the percentage of positive T cells for the indicated genes in the CD4 MLR **(C)**, and CD8 MLR **(D)**.

ICPs are important regulators of the immune system for which the MLR assay is often used to study their function. We performed flow cytometry and scRNA-seq analyses of ICPs expression by T lymphocytes over time in CD4 and CD8 MLRs. Flow cytometry indicated that immunoreactive T cells were characterized by higher mean fluorescent intensity (MFI) or percentage of expression of ICPs such as PD-1, TIM-3, ICOS and LAG-3 as well as the transcription factor TOX (**Figures 4A, B**; **Supplementary Figures S1**, **S2**, **S5**). The levels of expression of these ICPs increased over time in both CD4 and CD8 MLRs (**Supplementary Figure S5**). Interestingly, while the maximum percentage of expression of ICPs was similar between CD4^+^ and CD8^+^ T cells in the TCR-stimulated conditions, it remained lower in CD8 MLRs compared to CD4 MLRs, irrespective of the donor (**Supplementary Figure S5**).

These results were confirmed at the transcriptomic level using scRNA-seq data. Indeed, mRNAs encoding these ICPs were mainly detected in the Tcm, Treg and Thelper CD4^+^ T cells and Tcyt CD8^+^ T cells (**Figures 4C, D**). Of note, PDCD1 and TOX mRNA detections were sparser than other studied markers while having similar protein levels. This could be explained by post-transcriptional regulation. Indeed, it has been previously shown that PDCD1 mRNA turn-over is rapid in CD8^+^ T cells (45). Interestingly, PDCD1 and TOX seemed to be co-expressed in some CD8^+^ T cells but the number of cells that could hence be considered as exhausted T cells remained very low. The level of expression of ICPs mRNAs not only increased over time in a global manner in T cells (**Figures 4C, D**) but this increase could even be observed within the Tcm, Treg, Thelper and Tcyt subpopulations (**Supplementary Figure S6**). Thus, the increase in ICPs expression in MLR over time is not a simple consequence of cell composition evolution but a phenotype that strengthens over time in immunoreactive T cells in the MLR, without inducing a marked evolution toward T cell exhaustion.

**Figure 5:**
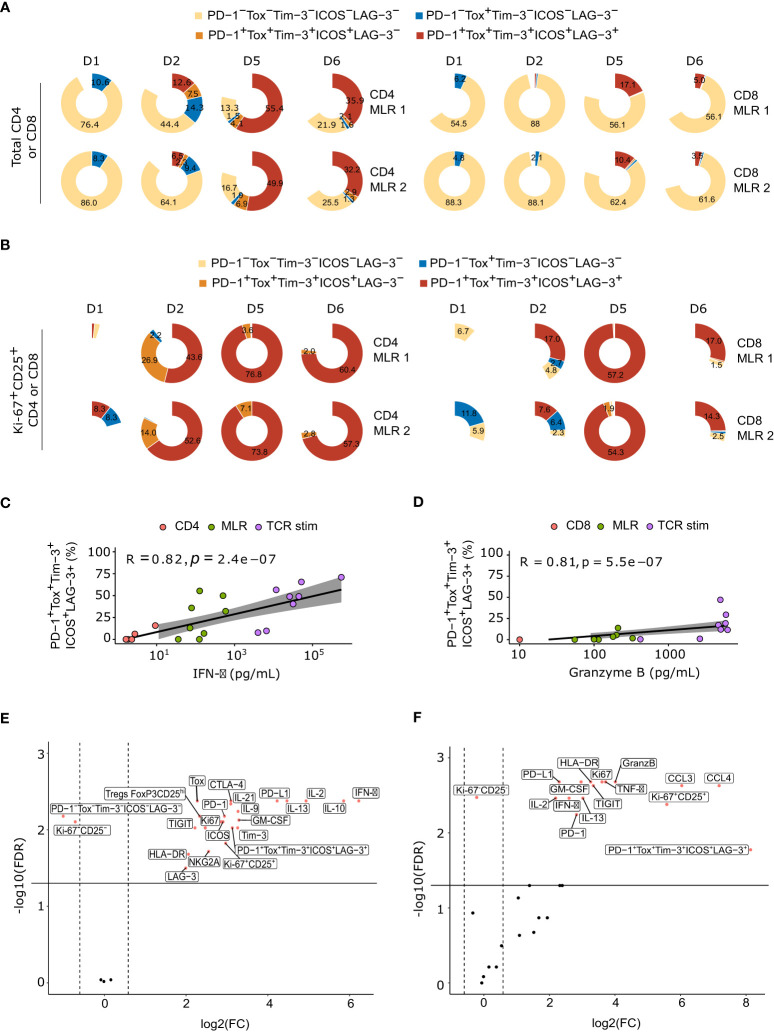
MLR-stimulated T cells are characterized by the co-expression of ICPs over time. **(A)** Pie plot of the percentage of ICPs co-expression at each timepoints in total CD4^+^ T cells (left panel) or total CD8^+^ T cells (right panel), for each donor. **(B)** Pie plot of the percentage of ICPs co-expression at each timepoints in Ki-67^+^CD25^+^ CD4^+^ T cells (left panel) or Ki-67^+^CD25^+^ CD8^+^ T cells (right panel), for each donor. **(C)** Spearman correlation between IFN-γ secretion and percentage of ICPs co-expression in CD4^+^ T cells. **(D)** Spearman correlation between granzyme B secretion and percentage of ICPs co-expression in CD8^+^ T cells **(E, F)** Volcano plots of Log10 transformed Wilcoxon rank-sum test P and the log10-transformed ratio according to soluble factors and flow cytometry markers of MLR-stimulated versus non-stimulated CD4^+^ T cells **(E)** or CD8^+^ T cells **(F)**. TCR stim: T cells stimulated with anti-CD3 and anti-CD28 antibodies.

We then explored the co-expression profile of all these ICPs by flow cytometry. MLR-stimulated T cells displayed both immune checkpoint activators (ICOS) and immune checkpoint inhibitors (PD-1, TIM-3, LAG-3) markers on their surface, as well as described exhaustion markers (TOX) (**Figure 5A**). This ICPs co-expressing population also increased over time, representing up to 52.6 +/- 2.7% and 9.4 +/- 4.2% of cells at day 5 in CD4 and CD8 MLRs, respectively. In particular, Ki-67^+^CD25^+^ immunoreactive lymphocytes T cells mainly co-expressed all these markers, especially at day 5 (**Figure 5B**). In addition, co-expression of ICPs significantly correlated with IFN-γ and GzmB releases, respectively in the CD4 and CD8 MLR (**Figures 5C, D**). We then performed an analysis of the most differentiating parameters between MLR-stimulated and non-stimulated T cells: ICPs co-expression was one of the best cell surface markers of MLR response in the CD4 MLR and IFN-γ appeared to be the top 1 secreted marker (**Figure 5E**). In CD8^+^ T cells, although the percentage of co-expressing ICPs is lower, it remains the main marker of MLR response, along with Ki-67-CD25 co-expression as well as CCL3, CCL4, GzmB and TNF-α secretions (**Figure 5F**). Consequently, upon MLR-stimulation, immunoreactive cells co-express ICPs, and this co-expression is closely linked to cytokine production.

### 3.5 All signals required for full T cell activation are detected between MoDCs and T cells in MLR

**Figure 6:**
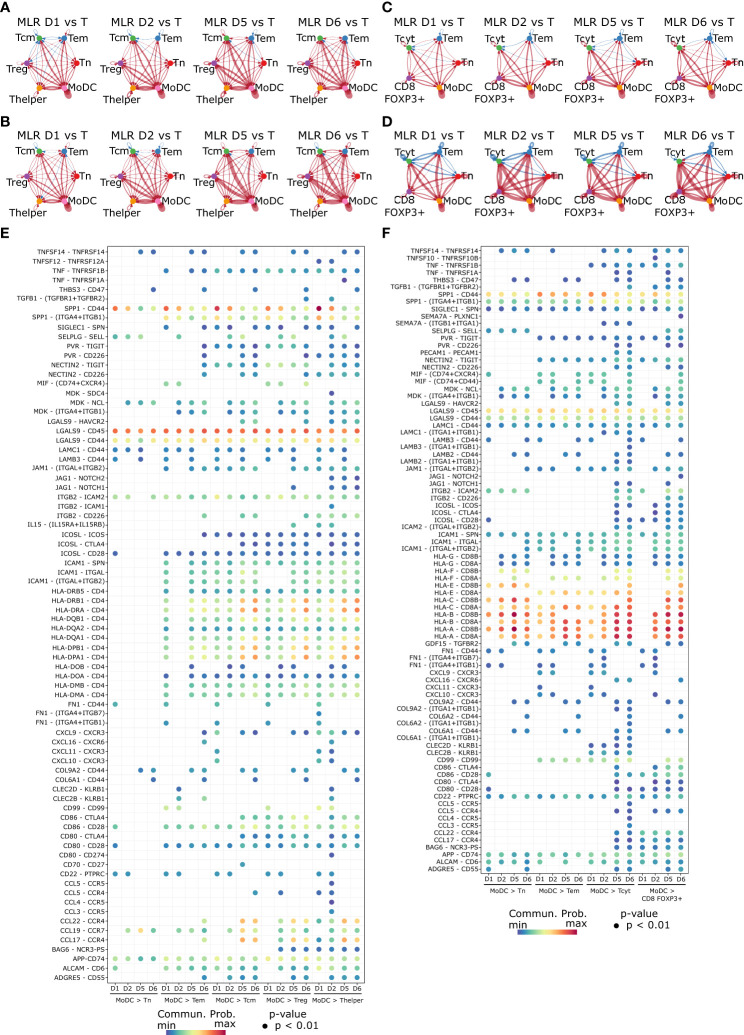
Cell-cell communication network inference highlights components of the immunological synapse occurring between MoDCs and T cells in the MLR. **(A–D)** Circle plot representing the differential number of interactions **(A)** or the interaction strength **(B)** in the cell-cell communication networks for the CD4 MLR, and CD8 MLR **(C, D)**. The width of edges represents the relative number of interactions or interaction strength. Red (or blue) colored edges represent increased (or decreased) signaling in the second dataset compared to the first one. **(E, F)** Bubble plot showing the ligand-receptor pairs involvement in MoDCs to T cells communication in the CD4 MLR **(E)**, and CD8 MLR **(F)**. Color represents the communication probability and size represents the p-value.

We explored T cell activation by non-self MoDCs in the MLR using cell-cell communication network inference from scRNA-seq data with CellChat. Quantification of differential numbers and strength of interactions between control non-stimulated T cells and MLR samples at different timepoints showed a predominant communication of MoDCs with nearly all T cells subtypes (**Figures 6A–D**). This crosstalk was globally increased at late timepoints in both CD4 and CD8 MLRs in terms of interaction numbers and strength.

We further investigated the specific ligand-receptor interactions among different cell populations in MLR over time by cell-cell communication network inference. In particular, in both CD4 and CD8 MLRs, MoDCs to T cells communication showed the three signals required for antigen-specific T cell activation (**Figures 6E, F**) (46–48). Signal 1, which involves antigen presentation by the MHC recognized by antigen-specific TCR, was depicted here by MHC II and TCR (HLA-DR – CD4) for the immunological synapse of CD4^+^ T cells, or by MHC I and TCR (HLA-ABC – CD8) for CD8^+^ T cells. Signal 2, which provides a co-stimulatory signal promoting T cell function and survival, was evidenced by the interaction of CD28 with CD80/86. Signal 3, which results from the secretion of cytokines, could be observed with IL15 – IL15RA/IL2RB interaction (particularly in the CD4 MLR). Other important elements of the immunological synapse were also observed here, such as integrins, which facilitate adhesion between cells, or chemokines (CCR/CCL and CXCR/CXCL), responsible for chemotaxis and recruitment of T cells by MoDCs. Finally, cell-cell communication analysis also exhibited markers of activation induced by the immunological synapse, such as TIGIT, Tim-3, ICOS and ICOS-L on Tcm, Thelper and Tcyt, as well as markers of allo-immune activated Tregs (APP – CD74). In brief, cell-cell communication analysis not only highlighted the main components of the immunological synapse in both CD4 and CD8 MLRs but also revealed some of the resulting T cell activation markers.

### 3.6 T cells activation signaling evolves over time

**Figure 7:**
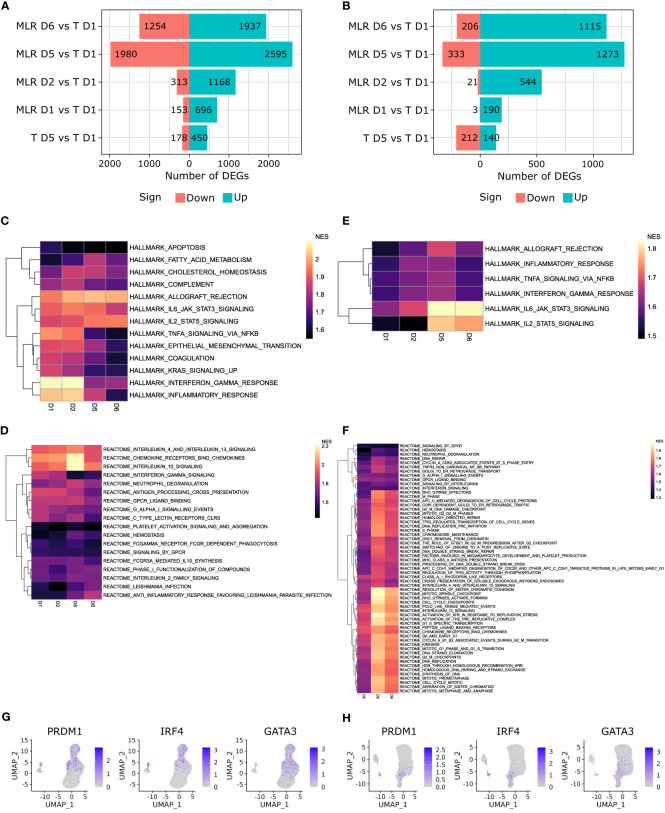
T cells activation signaling have differential kinetics between CD4 and CD8 MLRs. **(A, B)** Pyramid plot representing the number of DEGs over time in the CD4 MLR **(A)**, and CD8 MLR **(B)**. **(C, D)** Heatmap of NES for Hallmark gene sets enriched with adjusted p-value < 0.01 in MLR at all timepoints but not in non-stimulated T cells at day 5 *versus* day 1 in the CD4 MLR **(C)**, and CD8 MLR **(D)**. **(E, F)** Heatmap of NES for Reactome gene sets enriched with adjusted p-value < 0.05 in MLR at all timepoints but not in non-stimulated T cells at day 5 *versus* day 1 in the CD4 MLR **(E)**, and CD8 MLR **(F)**. **(G, H)** UMAP representation of indicated genes expression in the CD4 MLR **(G)**, and CD8 MLR **(H)**.

Downstream signaling of T cell activation was elucidated by pathway analysis on differentially expressed genes over time in MLR. As biological replicates of scRNA-seq data were available, we applied DESeq2 on count matrices aggregated to the sample level on T cell subsets. Paired non-stimulated T cells at day 1 were used as a reference for differential analysis. In both CD4 and CD8 MLRs, the number of differentially expressed genes increased drastically over time until day 5 before slightly decreasing at day 6 (**Figures 7A, B**). In control non-stimulated T cells samples, transcriptomic changes could also be observed between late and early timepoints, but they remained low compared to the MLR over the same period. Hence, T cell activation by MoDCs in the MLR had striking effects at the transcriptomic level.

Gene set enrichment analysis (GSEA) was run on Hallmark and Reactome gene sets to identify signaling pathways involved in T cell activation upon MLR. Several gene sets were enriched in MLR but not in non-stimulated T cells at day 5 *versus* day 1 (**Figures 7C–F**, **Supplementary Figure S7**, **Table S1**). We could observe different patterns of kinetics within enriched gene sets, defining early and late signaling pathways. In the CD4 MLR, Hallmark Inflammatory Response, Hallmark Interferon Alpha Response, Hallmark and Reactome Interferon Gamma Response, Hallmark TNFA Signaling via NFKB, Reactome Antigen Processing Cross Presentation gene sets had the highest normalized enrichment scores (NES) at early timepoints. This would indicate that the initial signals upon antigen-specific T cell activation involves the interferon and TNF-α signaling pathways. Then, Hallmark Allograft Rejection, Hallmark and Reactome IL2 Signaling, Reactome Chemokine Receptors Bind Chemokines, Reactome IL10 as well as IL4 and IL13 Signaling gene sets had the highest NES at late timepoints. In the CD8 MLR, the same signaling pathways were observed but they all had the highest NES at late timepoints. This is in line with the results presented above showing a weaker T cell responsiveness in the CD8 MLR. The up-regulation of multiple cytokine and chemokine signaling pathways once again highlights the polyfunctionality induced by MLR. To go further, we evaluated T cell polarization assessing gene expression patterns of common Th1/Tc1 and Th2/Tc2 transcription factors. We found that Th1/Tc1 markers PRDM1 and IRF4 as well as Th2/Tc2 marker GATA3 were up-regulated upon MLR stimulation (**Figures 7G, H**). Therefore, after early inflammation signaling, MLR stimulation leads to a specific signaling pathway, *i.e.* the allograft rejection, and implicates multiple polarizations and a polyfunctional response.

## 4 Discussion

Mixed Lymphocyte Reaction (MLR) is a widely used assay in drug discovery to model an allogeneic immune reaction and evaluate the immunomodulatory capacities of drug candidates. In most cases, MLR is carried out using total CD3^+^ T cells. The outcome of MLR is determined based on cytokine release and cell proliferation. With the aim of describing, better characterizing the kinetics of this model and refining the specificities of CD4^+^ and CD8^+^ T cells activation in this assay, we monitored phenotypic changes associated with immune response at the protein and transcriptomic levels in separated CD4 and CD8 MLRs using multiplex soluble factor analysis, high-dimensional flow cytometry and single cell RNA sequencing.

Among the different assays to evaluate T cell activation, MLR mimics the physiological antigen presentation to T cells via the recognition of an HLA mismatch. Compared to T cell activation assay using TCR-stimulation only, via anti-CD3 and/or anti-CD28 stimulation, MLR is particularly interesting as it engages the whole immunological synapse. Profiling of cytokines production as well as transcriptomic-based pathway analysis highlighted that MLR-stimulated cells are capable of secreting polyfunctional cytokines and that both CD4^+^ and CD8^+^ T cells acquire a phenotype presenting Th1 and Th2 as well as Tc1 and Tc2 polarization respectively. Secretion of cytokines is a good marker of T cell activation. As polyfunctionality is also observed in cancer patients, this phenomenon confirms the benefits of using this model for *in vitro* investigation of immunotherapy molecules (49, 50). Moreover, while TCR stimulation by CD3 and CD28 antibodies induced a strong activation of both CD4^+^ and CD8^+^ T cells from day 1 in this study, MLR presented a more gradual and moderate activation that probably better recapitulates physiological responses. Upon MLR stimulation, we could also observe Tregs that expanded and remained over time whereas this population was not detected in anti-CD3/anti-CD28 stimulated T cells. This suggests that MLR is a suitable model to evaluate effects of a compound on Tregs in total CD4^+^ T cell or Treg-sorted reaction.

Over the course of MLR, whether it is CD4 or CD8, we observed by flow cytometry the appearance of a specific immunoreactive population. We have shown that this population is predominantly activated and proliferative as evidenced by the expression of both Ki-67 and CD25 (40, 41). Single cell RNA sequencing analyses revealed that this population is mostly composed of Thelpers and Tregs for the CD4 MLR and mostly cytotoxic T lymphocytes for the CD8 MLR. Moreover, the Ki-67^+^CD25^+^ Thelpers, Tregs and Tcyts exhibited an up-regulation of T cell activation-related genes expression that could represent new potential markers of MLR response: PBK, LRR1 and MYO1G. PBK encodes for a mitotic serine/threonine kinase present in testis, placenta and germinal center and seems to support lymphoid cells activation (51, 52). LRR-1 protein acts as a regulator of the TNFRSF9/4-1BB stimulatory ICP activity, concomitantly to T cell proliferation and activation (53, 54). MYO1G is involved in T cell motility and plays a role in DC-T interaction to trigger efficient T cell engagement and activation (55). Intriguingly, we also found Ki-67^-^CD25^+^ cells in the CD4 MLR. Analysis of differentially expressed genes in this population showed the overexpression of interferon signaling pathway related genes. This pathway was associated with the early phase of activation. Hence, we can hypothesize that these cells are early activated rather than in the refractory phase. In this study, we could also observe by flow cytometry that the immunoreactive cells co-expressed the exhaustion marker TOX and most of the studied immune checkpoints upon activation, irrespective of their activatory or inhibitory effects. However, the number of cells that may present a T cell exhaustion phenotype by scRNA-seq remained very low and the percentage of cells co-expressing TOX and ICPs proteins did not stabilize over time but decreased at day 6. Therefore, in this assay, TOX and ICPs may be phenotypic markers of high activation rather than markers of exhaustion. Apart from the functional definition, *i.e.* the inability to produce cytokines and/or to kill targeted cells after repeated stimulations, the phenotypic characterization of exhaustion remains unclear. Some studies presented TOX and/or the co-expression of PD-1, Tim-3 and/or Lag-3 as the hallmark of T cell exhaustion, whereas they are also known to be expressed upon activation (56–58). Others have refined the definition by differentiating two subsets of exhausted T cells (progenitor exhausted T cells and terminally differentiated exhausted T cells) (59). Markers of exhaustion being also present upon activation, it is difficult to fully characterize T cell exhaustion. Overall, a more comprehensive definition of exhaustion is needed to differentiate exhausted T cells from highly activated ones (60). Consequently, while MLR assay is highly effective in recapitulating T cell activation, it does not appear to be an appropriate model to evaluate T cell exhaustion.

In both CD4 and CD8 MLRs, the proportion of immunoreactive cells expressing Ki-67^+^CD25^+^ and co-expressing ICPs correlated with cytokine release and ranked among the best markers of MLR-induced immune response. Nonetheless, the response amplitude was not the same between CD4 and CD8 MLRs. Whereas reactivity between CD4^+^ and CD8^+^ T cells was equivalent in TCR-activated T cells (treated with anti-CD3/anti-CD28 antibodies), cell expansion and the percentage of immunoreactive cells, expressing Ki-67^+^CD25^+^ and co-expressing ICPs, were lower in CD8 MLR compared to CD4 MLR. In this context, while MoDCs can induce a strong CD4^+^ T cell activation, cross-priming of naïve CD8^+^ T cell might be less effective for their activation, as previously described *in vivo* (61). To better understand these findings, we examined MoDC-T cell interactions over time and associated immunological pathways in T cells. Knowledge-based cell-cell communication network inference revealed similar patterns of interactions between CD4 and CD8 MLRs. Differences in reactivity amplitude do not appear to be due to the lack of a specific interaction between MoDCs and CD8^+^ T cells. We then evaluated the resulting signaling pathways involved in T cell activation. Here, CD4^+^ and CD8^+^ T cells presented different activation patterns. In the CD4 MLR, we observed that pathways related to acute inflammation were highly enriched at early timepoints, while the MLR-specific ones (*i.e.* allograft rejection) were more enriched at late timepoints. The CD8 MLR showed enrichments of both kinds of pathways but they all became highly enriched only at late timepoints. Hence, CD8^+^ T cells activation following interaction with MoDCs seems to be delayed and weaker compared to CD4^+^ T cells. A higher number of donors is needed to consolidate these observations. However, this major kinetic difference of activation between CD4 and CD8 MLRs should be taken into account when assessing immunotherapy drug candidates. The present study focused on CD4^+^- and CD8^+^-specific MLR assays. MLR conducted on total CD3^+^ T cells will be studied in the future as CD4^+^-CD8^+^ T cells interaction might impact activation patterns.

In conclusion, we have demonstrated that during MLR, following effective interaction of T cells with MoDCs, an initial inflammatory response is observed, involving the IFN-γ and TNF-α signaling pathways. This leads to further activation and proliferation of T cells, accompanied by the co-expression of both activatory and inhibitory immune checkpoints, the secretion of polyfunctional cytokines and the polarization towards both Th1/Tc1 and Th2/Tc2. We also identified PBK, LRR1 and MYO1G as new potential markers of T cell activation in the context of an allogeneic response. The separated analysis of CD4 and CD8 MLRs highlighted that this assay is a physiologically relevant tool to evaluate molecules targeting activation of CD4^+^ T cells including Tregs. However, caution should be taken for the assessment of CD8^+^ T cells late activation phase and/or exhaustion. Altogether, these results decipher the T cell responses observed in the widely used MLR assay, contributing to the improvement of immunomodulatory compounds’ evaluation for future therapeutic treatment.

## Data availability statement

The datasets presented in this study can be found in online repositories. The names of the repository/repositories and accession number(s) can be found below: https://www.ncbi.nlm.nih.gov/, GSE242428.

## Ethics statement

The studies involving humans were approved by Ethics committee of Etablissement Français du Sang (EFS), Ile-de-France (agreement n°21/EFS/035). The studies were conducted in accordance with the local legislation and institutional requirements. The participants provided their written informed consent to participate in this study.

## Author contributions

AM: Conceptualization, Data curation, Formal Analysis, Investigation, Methodology, Software, Validation, Visualization, Writing – original draft. AD: Conceptualization, Data curation, Formal Analysis, Investigation, Methodology, Validation, Visualization, Writing – original draft, Software. EB: Methodology, Writing – original draft, Supervision, Validation. SD-C: Investigation, Writing – original draft. MF: Investigation, Writing – original draft. NP: Supervision, Writing – original draft. DW: Supervision, Writing – original draft. HD: Methodology, Supervision, Writing – original draft. J-PG: Funding acquisition, Methodology, Supervision, Conceptualization, Writing – original draft. CL: Methodology, Supervision, Conceptualization, Writing – original draft. VB: Conceptualization, Funding acquisition, Supervision, Writing – original draft. VL: Conceptualization, Funding acquisition, Methodology, Supervision, Writing – original draft.

## Supplementary material

Supplementary Figure 1 Expression of each flow cytometry markers mean fluorescent intensity in CD4 MLRs. **(A)** Time-course concentrations of secreted cytokines for each donor in CD4 MLR. Each dot and bars represent the mean and standard deviation (SD) in MLR-stimulated T cells for each soluble factor at the corresponding time. **(B)** Flow cytometry UMAP dimensionality reduction representation of CD4^+^ T cells upon MLR stimulation colored by indicated markers mean fluorescent intensity for each donor.

Supplementary Figure 2 Expression of each flow cytometry markers mean fluorescent intensity in CD8 MLRs. **(A)** Time-course concentrations of secreted cytokines for each donor in CD8 MLR. Each dot and bars represent the mean and standard deviation (SD) in MLR-stimulated T cells for each soluble factor at the corresponding time. **(B)** Flow cytometry UMAP dimensionality reduction representation of CD8^+^ T cells upon MLR stimulation colored by indicated markers mean fluorescent intensity for each donor.

Supplementary Figure 3 Signature genes of single cell RNA-seq clusters. **(A, B)** Bubble plot showing expression of representative signature genes of the CD4 MLR **(A)**, and CD8 MLR **(B)**. Color represents the normalized expression level and size represents the expression frequency.

Supplementary Figure 4 Detailed cell composition analysis for each donor. **(A)** Barplot of cells proportions colored by detailed phenotype in the CD4 MLR. **(B, C)** Barplot of cells proportions colored by main phenotype **(B)**, detailed phenotype **(C)** for donor 1 in the CD4 MLR. **(D, E)** Same as **(B)** and **(C)** but for donor 2. **(F, G)** Barplot of MKI67-IL2RA co-expression proportions by main phenotype for donor 1 **(F)**, donor 2 **(G)** in the CD4 MLR. **(H-N)** Same as **(A-G)** but for the CD8 MLR.

Supplementary Figure 5 PD-1 and Tim-3 are mainly expressed by T cells upon MLR stimulation and their expressions increase over time. **(A-C)** in MLR-stimulated CD4^+^ T cells, flow cytometry dot plot of PD-1 and Tim-3 expression in at day 1 and day 5 per donor **(A)** and corresponding histograms **(B)**, PD-1 (left panel) or Tim-3 (right panel) expression at each day for unstimulated T cells (open circles), MLR-stimulated T cells (closed circles) or TCR-stimulated T cells (open triangle) per donor (light green and dark green) **(C)**. **(D-F)** in MLR-stimulated CD8^+^ T cells, flow cytometry dot plot of PD-1 and Tim-3 expression in at day 1 and day 5 per donor **(D)** and corresponding histograms **(E)**, PD-1 (left panel) or Tim-3 (right panel) expression at each day for unstimulated T cells (open circles), MLR-stimulated T cells (closed circles) or TCR-stimulated T cells (open triangle) per donor (light blue and dark blue) **(F)**. TCR stim: T cells stimulated with anti-CD3 and anti-CD28 antibodies.

Supplementary Figure 6 ICPs expressions increase over time in immunoreactive T cells upon MLR stimulation. **(A, B)** Follow up of the percentage of positive cells for the indicated genes and populations in the CD4 MLR **(A)**, and CD8 MLR **(B)**.

Supplementary Figure 7 Pseudo-bulk RNA-seq samples clustering and extended heatmaps from pathway analysis. **(A, B)** PCA plot of pseudo-bulk RNA-seq samples in the CD4 MLR **(A)**, and CD8 MLR **(B)**. **(C, D)** Heatmap showing clustering of pseudo-bulk RNA-seq samples in the CD4 MLR **(C)**, and CD8 MLR **(D)**. **(E, F)** Heatmap of NES for Hallmark gene sets enriched in MLR but not in non-stimulated T cells at day 5 *versus* day 1 in the CD4 MLR **(E)**, and CD8 MLR **(F)**. Grey color corresponds to non-significant result with adjusted p-value > 0.01.
