# Supplementary figures and images for: Characterization of CD4+ and CD8+ T cells responses in the mixed lymphocyte reaction by flow cytometry and single cell RNA sequencing
Source: Front Immunol. 2024 Jan 12;14:1320481. doi: 10.3389/fimmu.2023.1320481 (PMC10820991; doi:10.3389/fimmu.2023.1320481)

A.

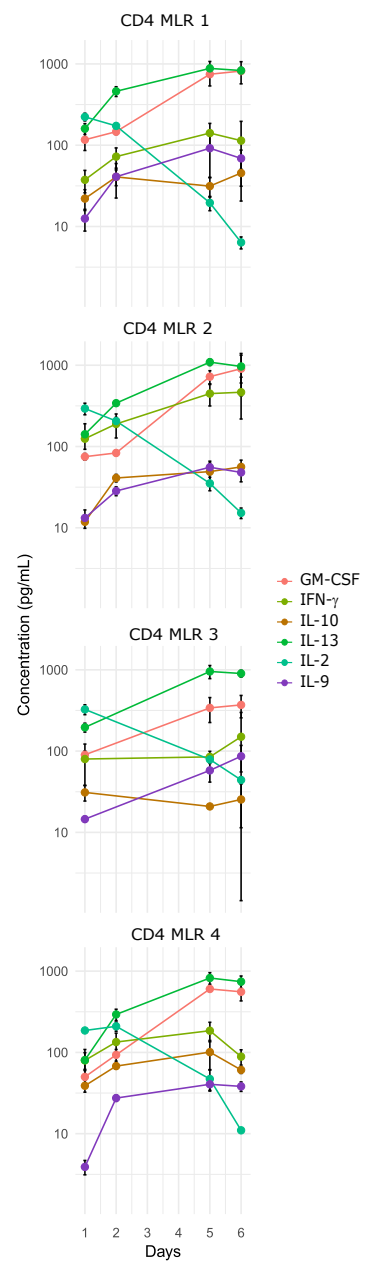

B.

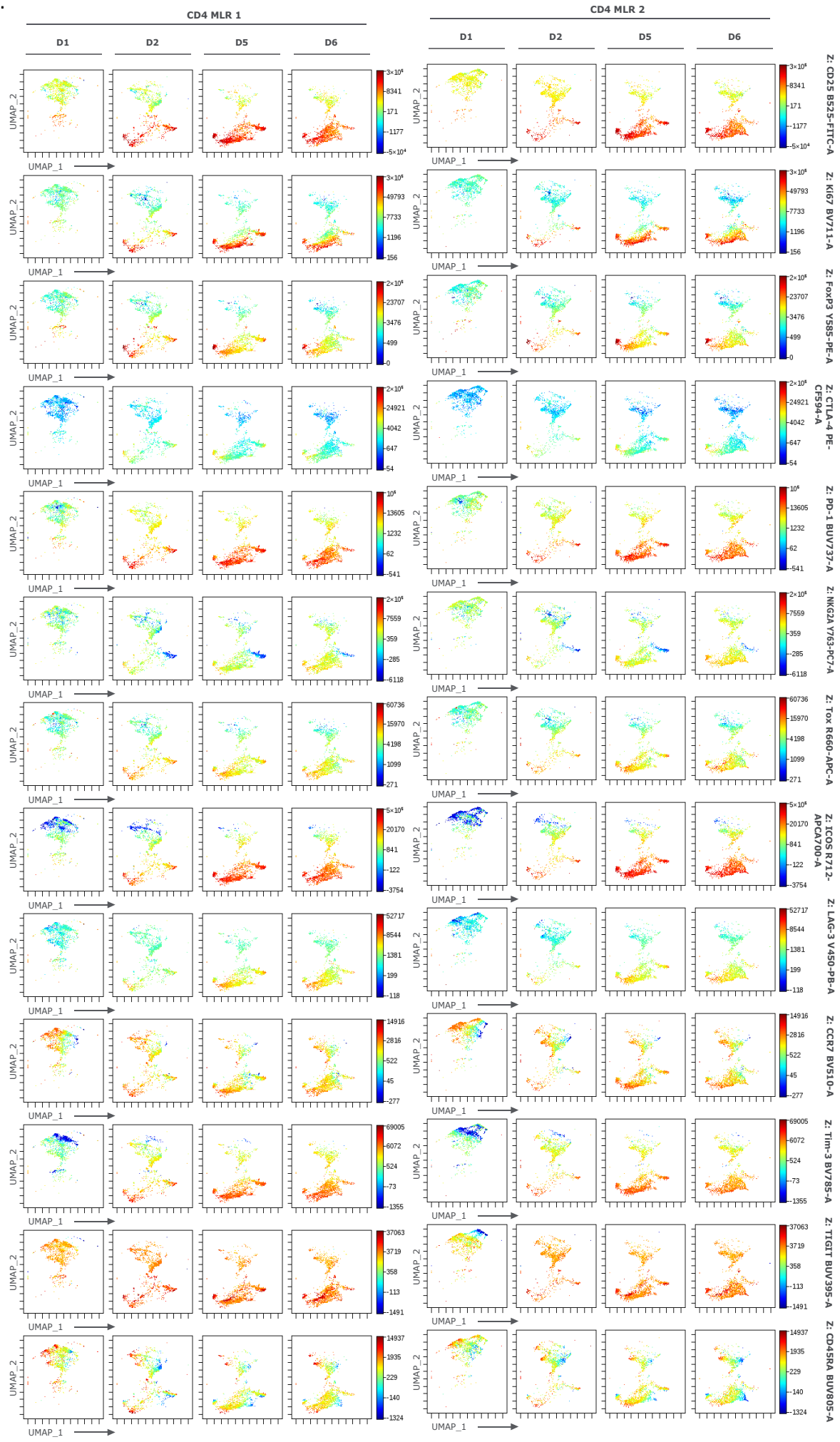

Figure S1

Supplement: Supplementary Figure 1 — Expression of each flow cytometry markers mean fluorescent intensity in CD4 MLRs. (A) Time-course concentrations of secreted cytokines for each donor in CD4 MLR. Each dot and bars represent the mean and standard deviation (SD) in MLR-stimulated T cells for each soluble factor at the corresponding time. (B) Flow cytometry UMAP dimensionality reduction representation of CD4+ T cells upon MLR stimulation colored by indicated markers mean fluorescent intensity for each donor. [file Image_1.pdf]

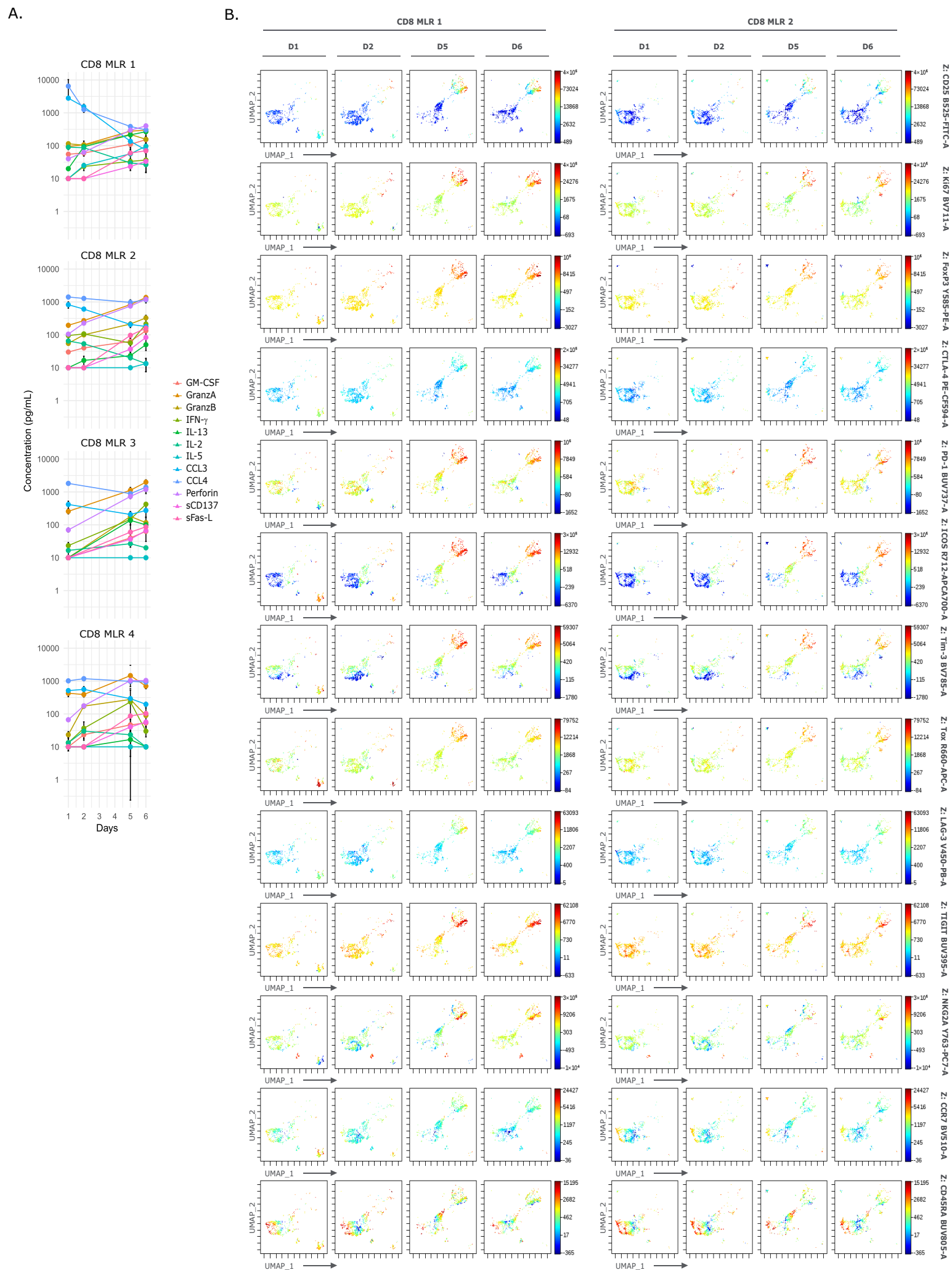

Figure S2

Supplement: Supplementary Figure 2 — Expression of each flow cytometry markers mean fluorescent intensity in CD8 MLRs. (A) Time-course concentrations of secreted cytokines for each donor in CD8 MLR. Each dot and bars represent the mean and standard deviation (SD) in MLR-stimulated T cells for each soluble factor at the corresponding time. (B) Flow cytometry UMAP dimensionality reduction representation of CD8+ T cells upon MLR stimulation colored by indicated markers mean fluorescent intensity for each donor. [file Image_2.pdf]

A.

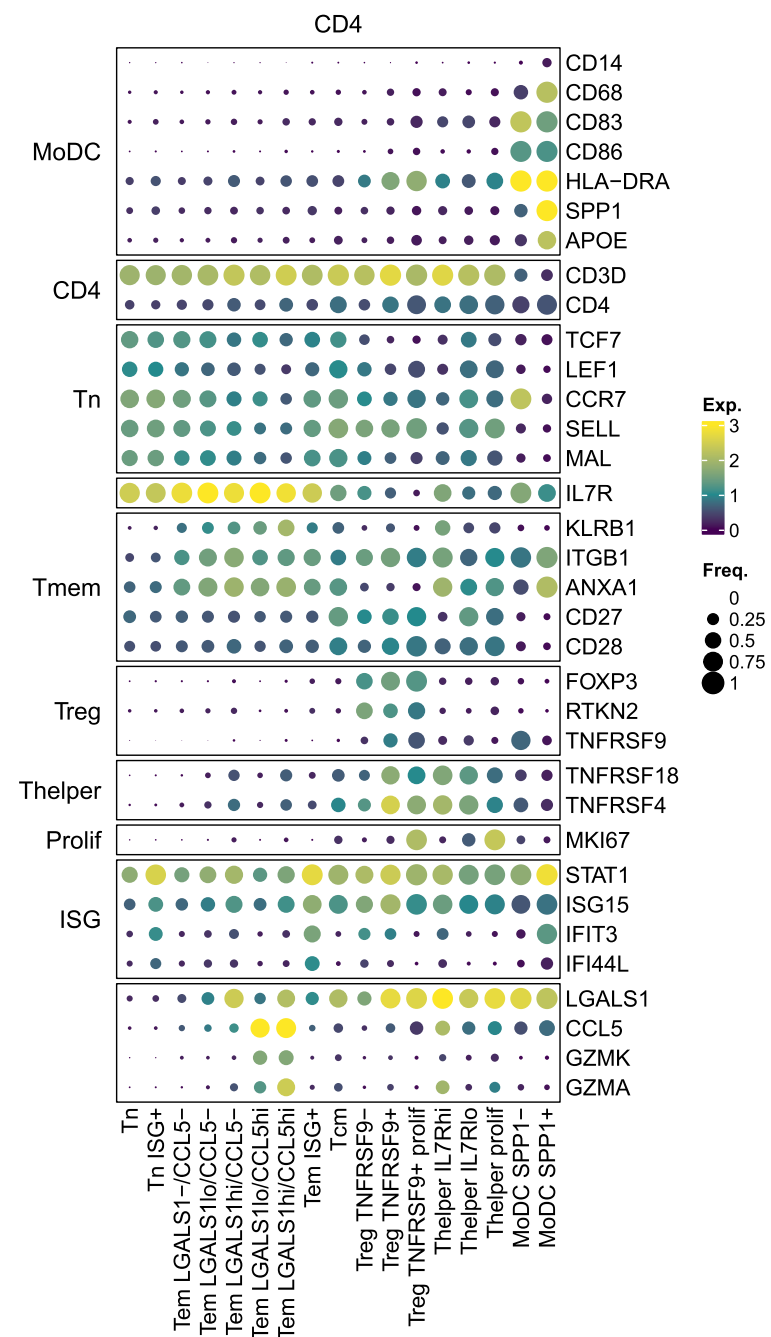

B.

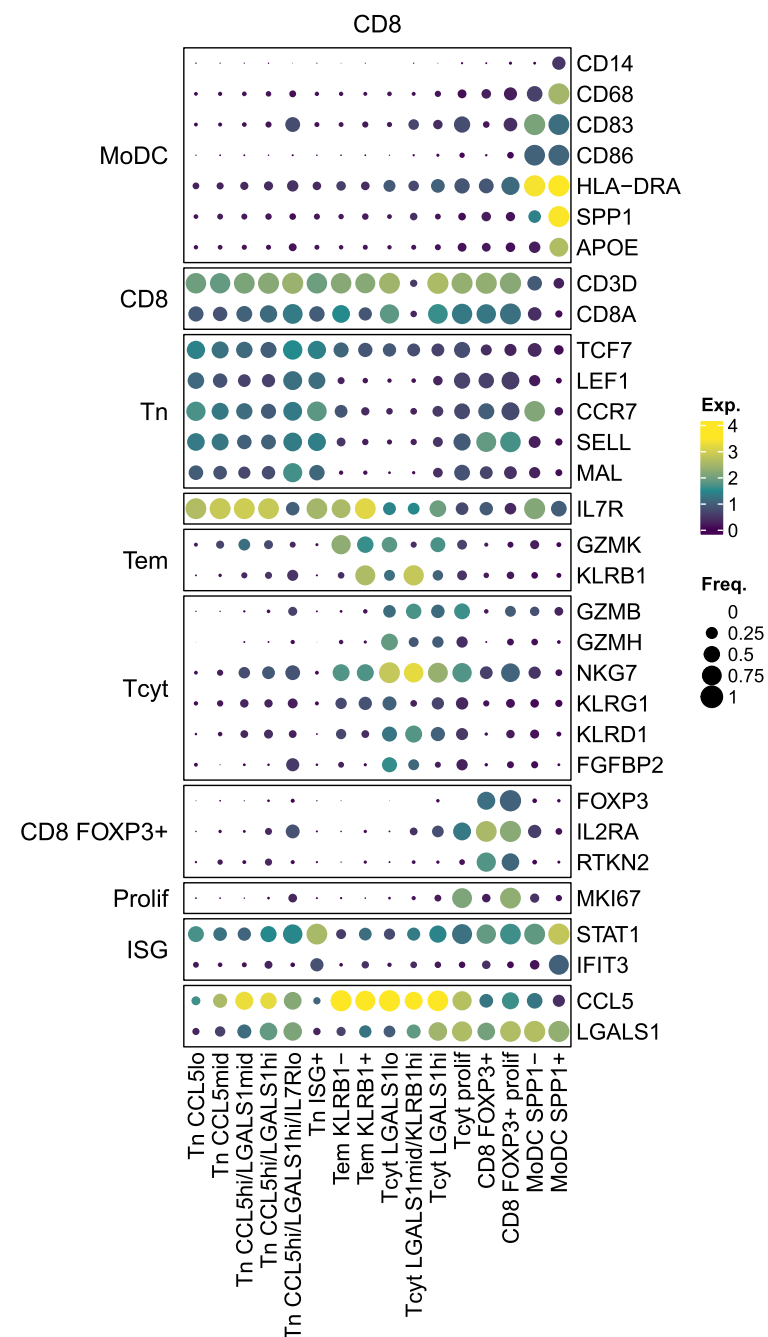

Figure S3

Supplement: Supplementary Figure 3 — Signature genes of single cell RNA-seq clusters. (A, B) Bubble plot showing expression of representative signature genes of the CD4 MLR (A), and CD8 MLR (B). Color represents the normalized expression level and size represents the expression frequency. [file Image_3.pdf]

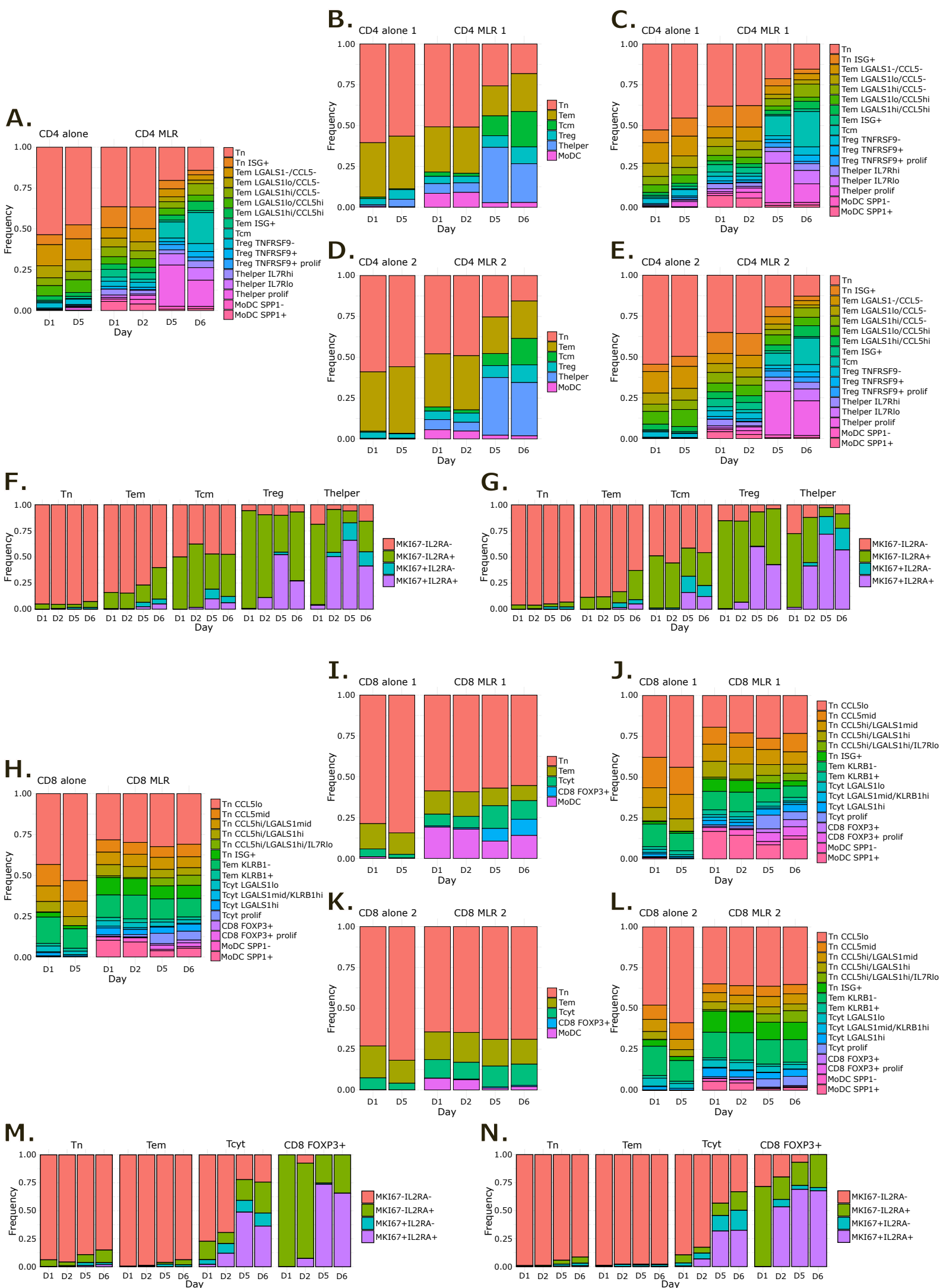

Figure S4

Supplement: Supplementary Figure 4 — Detailed cell composition analysis for each donor. (A) Barplot of cells proportions colored by detailed phenotype in the CD4 MLR. (B, C) Barplot of cells proportions colored by main phenotype (B), detailed phenotype (C) for donor 1 in the CD4 MLR. (D, E) Same as (B) and (C) but for donor 2. (F, G) Barplot of MKI67-IL2RA co-expression proportions by main phenotype for donor 1 (F), donor 2 (G) in the CD4 MLR. (H-N) Same as (A-G) but for the CD8 MLR. [file Image_4.pdf]

A.

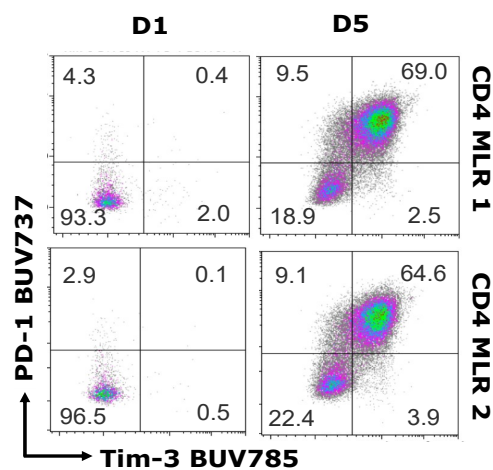

B.

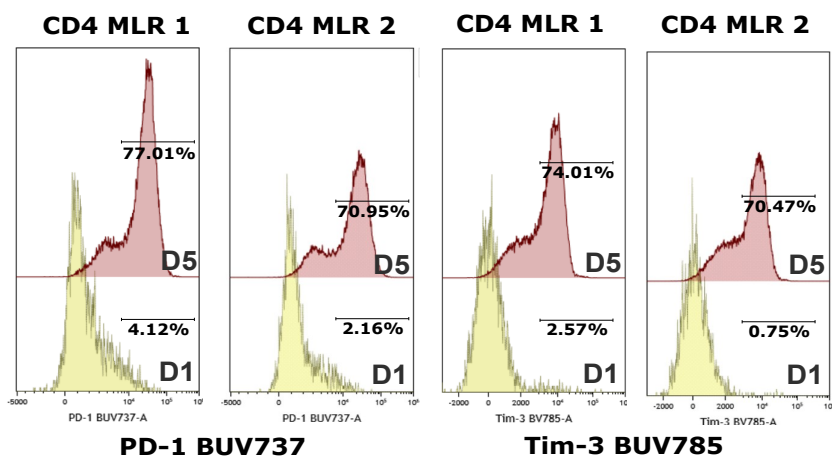

C.

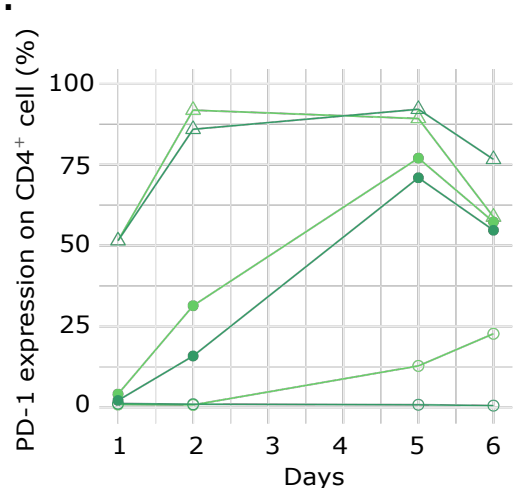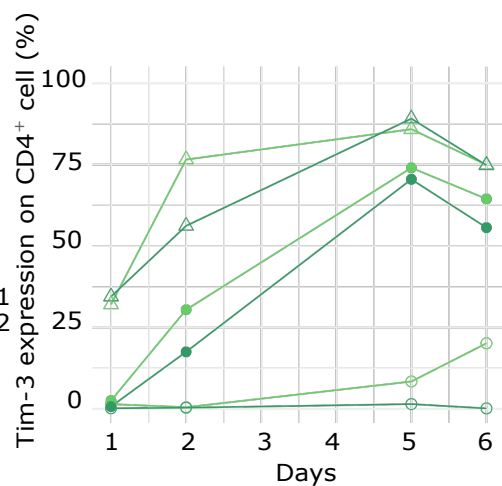

D.

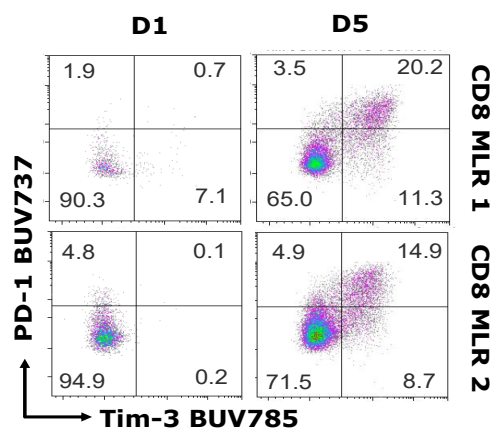

E.

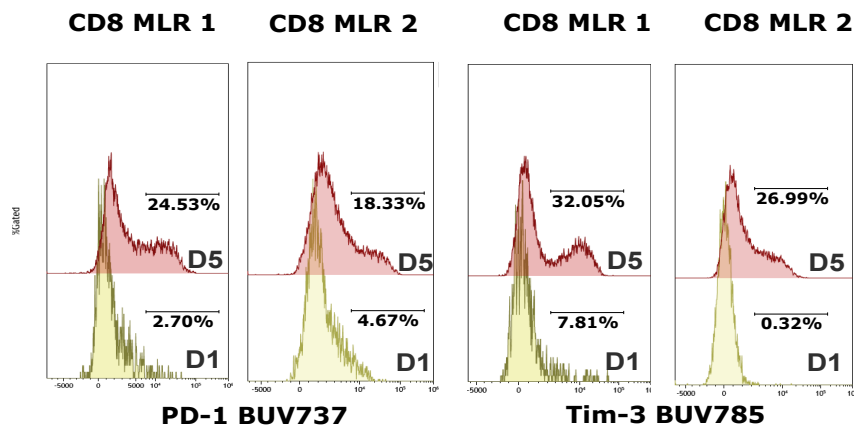

F.

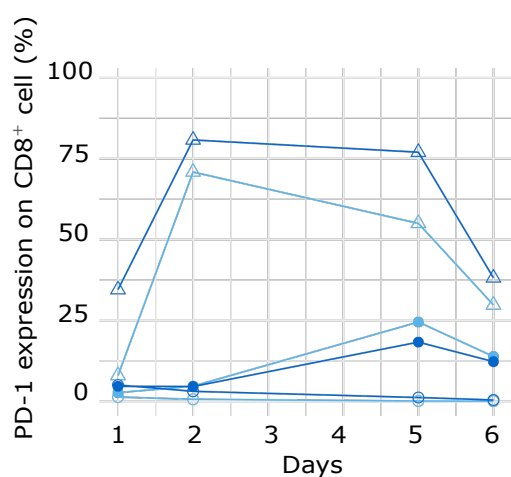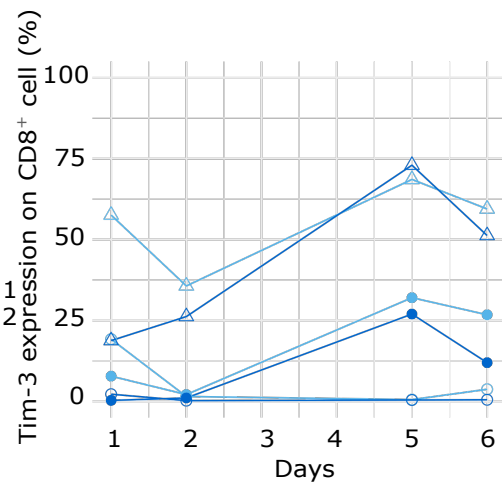

Figure S5

Supplement: Supplementary Figure 5 — PD-1 and Tim-3 are mainly expressed by T cells upon MLR stimulation and their expressions increase over time. (A-C) in MLR-stimulated CD4+ T cells, flow cytometry dot plot of PD-1 and Tim-3 expression in at day 1 and day 5 per donor (A) and corresponding histograms (B), PD-1 (left panel) or Tim-3 (right panel) expression at each day for unstimulated T cells (open circles), MLR-stimulated T cells (closed circles) or TCR-stimulated T cells (open triangle) per donor (light green and dark green) (C). (D-F) in MLR-stimulated CD8+ T cells, flow cytometry dot plot of PD-1 and Tim-3 expression in at day 1 and day 5 per donor (D) and corresponding histograms (E), PD-1 (left panel) or Tim-3 (right panel) expression at each day for unstimulated T cells (open circles), MLR-stimulated T cells (closed circles) or TCR-stimulated T cells (open triangle) per donor (light blue and dark blue) (F). TCR stim: T cells stimulated with anti-CD3 and anti-CD28 antibodies. [file Image_5.pdf]

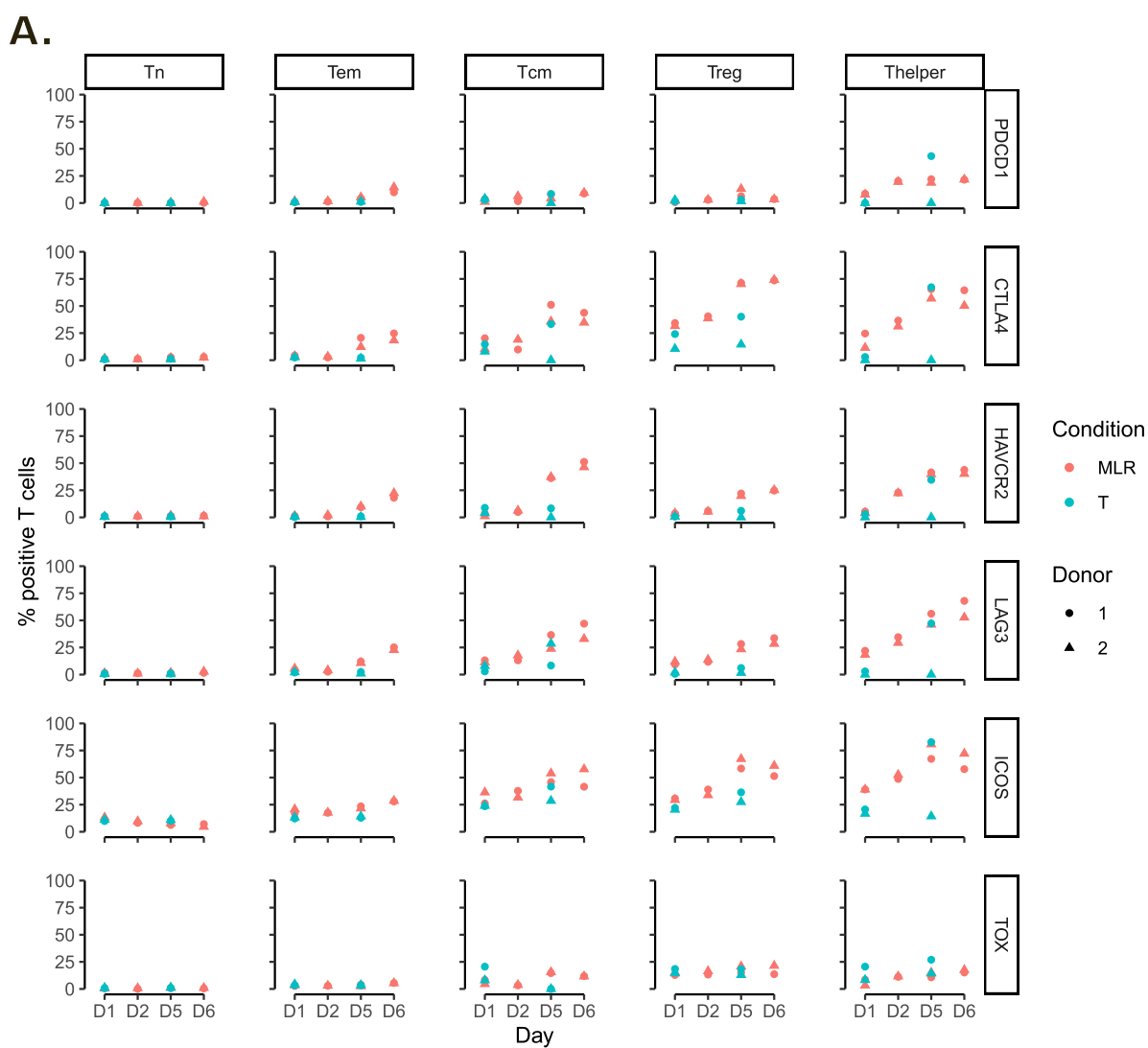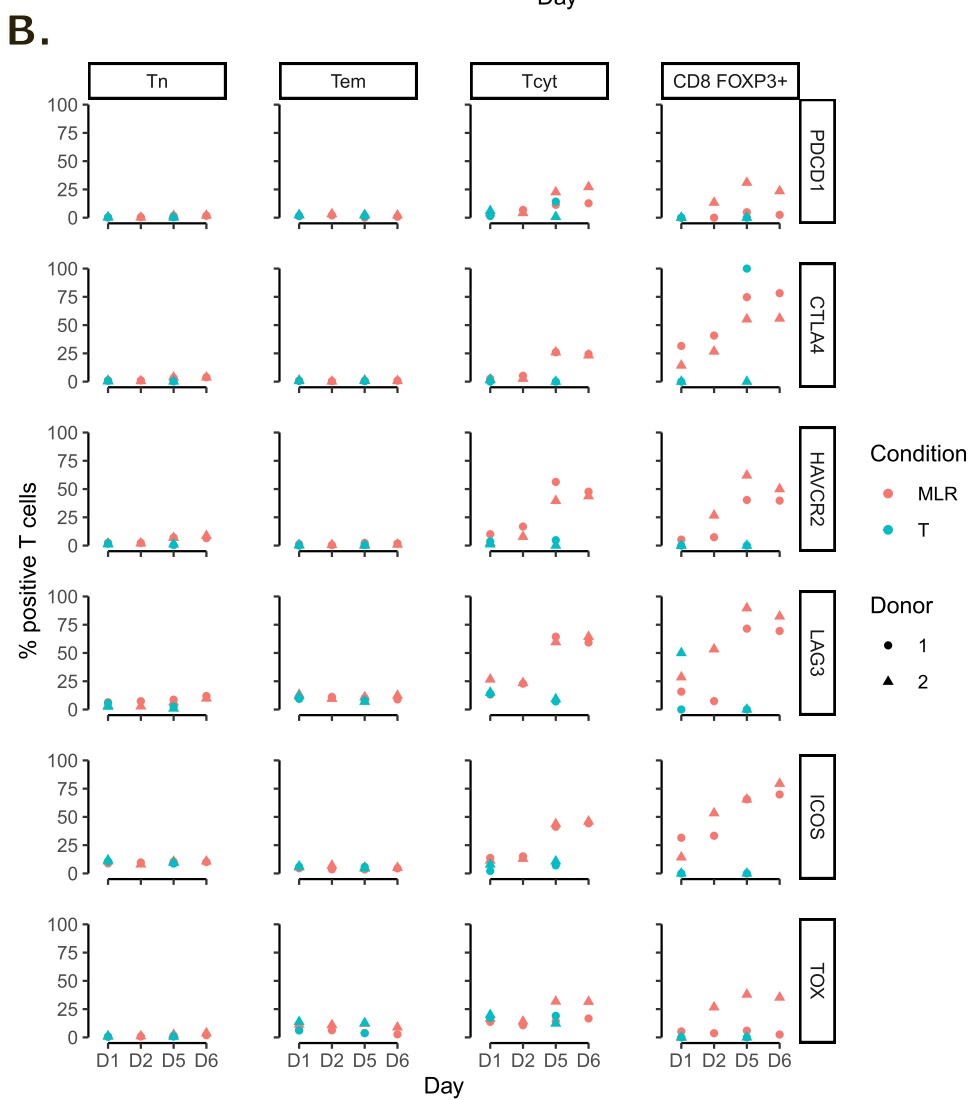

**Figure S6**

Supplement: Supplementary Figure 6 — ICPs expressions increase over time in immunoreactive T cells upon MLR stimulation. (A, B) Follow up of the percentage of positive cells for the indicated genes and populations in the CD4 MLR (A), and CD8 MLR (B). [file Image_6.pdf]

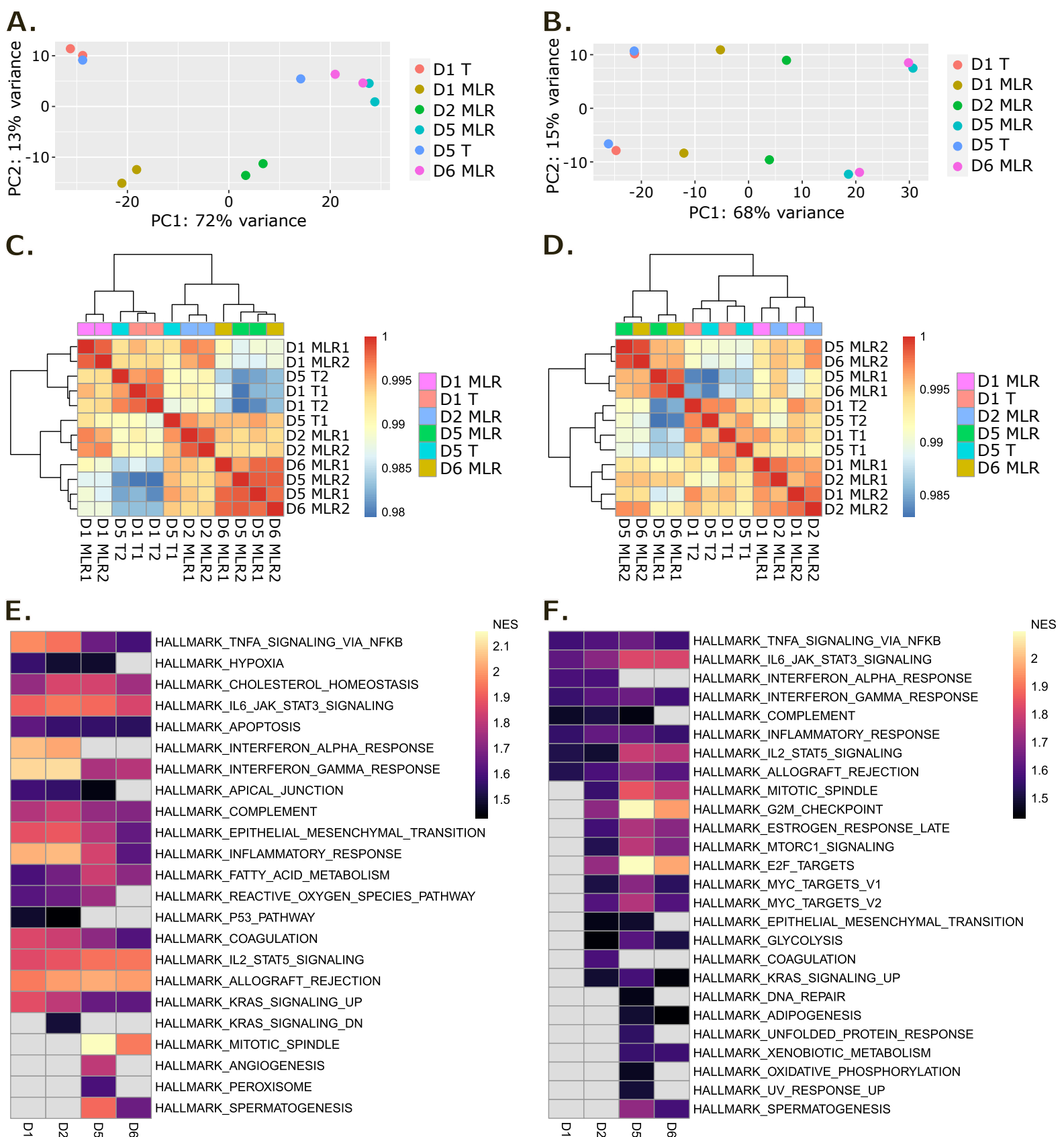

Figure S7

Supplement: Supplementary Figure 7 — Pseudo-bulk RNA-seq samples clustering and extended heatmaps from pathway analysis. (A, B) PCA plot of pseudo-bulk RNA-seq samples in the CD4 MLR (A), and CD8 MLR (B). (C, D) Heatmap showing clustering of pseudo-bulk RNA-seq samples in the CD4 MLR (C), and CD8 MLR (D). (E, F) Heatmap of NES for Hallmark gene sets enriched in MLR but not in non-stimulated T cells at day 5 versus day 1 in the CD4 MLR (E), and CD8 MLR (F). Grey color corresponds to non-significant result with adjusted p-value > 0.01. [file Image_7.pdf]
